# Bridged h‐BN Nanosheets Coatings: Simultaneous Shielding Atomic‐Oxygen Irradiation and Achieving Superior Friction Performance

**DOI:** 10.1002/advs.76093

**Published:** 2026-06-12

**Authors:** Zhuoyi Li, Changning Bai, Xingkai Zhang, Yusheng Liang, Wei Wu, Dingrui Zhou, Tao Du, Chunjin Wang

**Affiliations:** ^1^ State Key Laboratory of Ultra‐precision Machining Technology Department of Industrial and Systems Engineering The Hong Kong Polytechnic University Hong Kong China; ^2^ State Key Laboratory of Solid Lubrication Lanzhou Institute of Chemical Physics Chinese Academy of Sciences Lanzhou China; ^3^ College of Civil Engineering and Mechanics Lanzhou University Lanzhou China; ^4^ Key Lab of Smart Prevention and Mitigation of Civil Engineering Disasters of the Ministry of Industry and Information Technology Harbin Institute of Technology Harbin China

**Keywords:** atomic oxygen resistance, friction, functionalization, h‐BN nanosheets, polyvinyl alcohol

## Abstract

To confront extreme space environments, a combination of inorganic and organic coatings can provide a pathway for offering highly durable radiation resistance and anti‐friction simultaneously. Herein, a strong interfacial 3D hydrogen‐bonding network is designed to create robust hexagonal boron nitride (h‐BN) based polymer coatings with excellent mechanical and friction performance after atomic oxygen (AO) irradiation. The h‐BN‐based polymer coatings possess substantial improvements in structural stability, thermal conductivity (5.75 W·m^−1^·K^−1^, 24 times higher than pure organic coatings), tensile strength (48.1 MPa, improved by ∼ 200%), and low friction (0.12–0.14, reduced by ∼ 2.5 times) after AO irradiation. Notably, the oxygen transmission rate decreases from 2.53 to 0.35 cm^3^·m^−2^·24 h^−1^, displaying superior oxygen substance barrier performance. This exceptional performance arises from h‐BN providing dual physical and chemical barriers through its layered structure and chemical stability, effectively resisting AO irradiation. Additionally, the 3D network facilitates efficient load transfer and heat dissipation for coating structure stability because the concentrated load is instantly transmitted across the entire h‐BN surface, and the high thermal conductivity of h‐BN is exerted to prevent localized overheating. This work provides a design strategy for next‐generation radiation‐resistant materials that unify high thermal conductivity, robust mechanical and friction performance.

## 1 Introduction

In low Earth orbit (LEO) and deep‐space environments, protective equipment that combines exceptional mechanical strength, highly radiation resistance, low‐friction movement during deployment and retraction, and lightweight characteristics is of paramount importance for guaranteeing operational stability and safety [1, 2, 3, 4, 5]. Polymer coatings play an indispensable role to integrate these multi‐layer functions, due to its flexible molecular design and interfacial control capabilities [6, 7]. However, the physical and chemical attack of atomic oxygen (AO) on polymers in the LEO environment poses substantial challenges to the long‐term durability and results in the premature failure of shuttle missions. For instance, under the interconnected processes of energy and charge exchange, the mass loss and the erosion depth of polyimide are proportional to the AO flows [8, 9, 10, 11]. Providing a long spacecraft service life calls for increasing polymer resistance to AO, in which a way to solve the problem is the introduction of nanomaterials into polymer coatings [12, 13, 14, 15]. Incorporating nanomaterials can not only augment specific AO resistance of the polymer coating itself but also bring unexpected new characteristics.

The AO (∼5 eV) attack on a polymer changes its surface topography, thermal stability, and physical‐mechanical properties. The change in the surface topography and roughness affects the processes of momentum and energy transfer from the gaseous components of the Earth's atmosphere to spacecraft. The deterioration of mechanical properties and thermal management is hard to maintain stable operation and long‐term durability of the rotor [16]. Therefore, 2D nanomaterials (e.g., graphene and molybdenum disulfide), the coupling of high surface‐to‐volume ratios, thermal conductivity, excellent lubrication, and great mechanical properties, have emerged as promising filler candidates [17, 18, 19, 20, 21]. With the assistance of 2D nanomaterials, the erosion and depolymerization of polymer coatings caused by free radical chain reactions are weakened, and the diffusion pathways of AO are blocked. The governing process of AO‐induced polymer oxidative degradation and the physical or chemical adsorption is controlled, ensuring the structural stability of polymer coatings [22]. On the other hand, 2D layered structures showed their great potential to improve the friction and wear of polymer coatings, enhancing the smooth operation of the components. During sliding, it may act both as shearing films and nano‐roller bearings, thus potentially changing the friction mode from sliding to rolling friction, thus reducing friction losses [23, 24]. However, the amount of filler that can be incorporated into the polymer coating is limited, as excessively high filler content can compromise the flexibility of the polymer matrix and induce the generation of structural defects, serving as stress concentration sites and phonon scattering centers in heat transfer [25, 26, 27, 28]. Consequently, it is essential to design fillers that simultaneously possess both good dispersion and high concentration to achieve the optimization of AO shielding and friction in polymer‐based composites.

Hexagonal boron nitride (h‐BN), a typical 2D ceramic with honeycomb hexagonal lattices, has emerged as an exceptionally effective filler for radiation‐resistant and anti‐friction applications due to its unique combination of high thermal conductivity (400 W m^−1^ K^−1^), excellent electrical insulation, chemical inertness, and thermal stability [29, 30, 31, 32, 33]. Structurally, the diameter of atomic oxygen (∼304 pm) is larger than the lattice constant of h‐BN (∼250.4 pm), meaning it cannot directly penetrate the h‐BN honeycomb surface. Energetically, AO‐induced oxidation of h‐BN to boron oxide requires breaking two B‐N bonds (∼15‐20 eV), comparable to cleaving two C─C bonds in graphene, reflecting its high stability [34, 35, 36, 37]. In the tribological context, h‐BN works better under humid and high‐temperature conditions due to its stronger oxidation resistance and thermal management, further making it suitable as AO shielding material [38, 39, 40, 41, 42]. Exerting the anisotropic nature of h‐BN, the improvement of h‐BN orientation orderliness and dispersion would enhance the interaction probability between AO incident direction and h‐BN through‐plane direction [43]. However, conventional methods such as organic modification present inherent limitations, including excessive stacking, agglomeration, and limited filling capacity [44, 45, 46]. The precise control of process parameters and the need for directional adjustment are also crucial in this process. Therefore, the precise and controllable dispersion of hexagonal boron nitride (h‐BN) in polymer coatings, along with its high‐content filling and enhanced compactness, plays a crucial role in delaying the erosion of AO.

To address this challenge, we have developed a 3D hydrogen‐bonding network is designed to create robust h‐BN‐based polymer coatings to meet the requirement of achieving excellent mechanical and friction performance after AO irradiation. By employing ball milling, the amino groups were grafted onto the surface of h‐BN to enhance adhesion to PVA chains, akin to molecular anchors that firmly connect the components. This was followed by a UV irradiation/heat cross‐linking process to produce an h‐BN‐based polymer coating. The adhesion of h‐BN to PVA effectively mitigated aggregation, leading to a uniform and even distribution and high‐content filling of h‐BN throughout the coating. Our results demonstrate that the coating exhibits a thermal conductivity of 5.75 W·m^−1^·K^−1^ (a 24‐fold increase compared to pure PVA), an initial decomposition temperature of 269.3°C, a friction coefficient of 0.12–0.14, and a tensile strength of 48.1 MPa, improving the thermal stability, wear resistance, and mechanical performance. Furthermore, the oxygen transmission rate (OTR) of the advanced composite coating decreased significantly from 2.53 to 0.35 cm^3^·m^−2^·24 h^−1^, enhancing its gas barrier performance. DFT calculations and MD simulations further confirm that h‐BN improves interfacial bonding and enhances AO shielding, making it a promising candidate for protective coatings in extreme space environments. This work provides an innovative approach for the design of spacecraft protective coatings, with the potential to significantly enhance the long‐term stability and durability of materials under space conditions, thereby meeting the needs of future space missions requiring high‐performance materials.

## 2 Experiment Section

### 2.1 Materials

Hexagonal boron nitride (h‐BN, 99.99%, Shanghai Macklin Biochemical Co., Ltd.), acrylamide (AAm, Mw = 71.08 g mol^−1^, analytical grade, Chengdu Kelong Chemical Co., Ltd.), poly(vinyl alcohol) (PVA, degree of polymerization = 1750 ± 50, content≥99.0%, Sinopharm Chemical Reagent Co., Ltd.), polyvinylpyrrolidone (PVP, analytical grade, Tianjin Kemiou Chemical Reagent Co., Ltd.), N,N'‐methylenebisacrylamide (MBAA, Mw = 154.17 g mol^−1^, analytical grade, Chengdu Kelong Chemical Co., Ltd.), boric acid (Mw = 61.83 g mol^−1^, analytical grade, Lanzhou Shuangshuang Chemical Reagent Co., Ltd.), glutaraldehyde (Mw = 100.12 g mol^−1^, 50 wt.%, Chengdu Kelong Chemical Co., Ltd.), and ammonium persulfate (APS, Mw = 228.20 g mol^−1^, analytical grade, Chengdu Kelong Chemical Co., Ltd.) were used as received.

### 2.2 Fabrication of BNNS@AAm

h‐BN powder was dispersed in deionized water and frozen, and subsequently thawed under ultrasonic treatment. This freeze‐thaw cycle was repeated five times to achieve effective exfoliation and uniform dispersion. The suspension was then centrifuged at 1500 rpm for 10 min, after which the supernatant was collected and dried at 60°C in a forced‐air oven to obtain h‐BN nanosheets (BNNS). Separately, AAm was dissolved in deionized water to prepare an AAm solution. BNNS and AAm were mixed at a 1:1 mass ratio, followed by the addition of boric acid (5 wt.% of the total raw material mass). The mixture was subjected to ball milling at 450 rpm for 6 h under an argon atmosphere, with the temperature maintained below 40°C. The suspension was washed three times with acetone, and then the precipitate was collected by centrifugation. Finally, the AAm modified BNNS powder (BNNS@AAm) was obtained by vacuum drying at 60°C for 12 h.

### 2.3 Fabrication of Coating

To investigate the effect of BNNS@AAm loading, the coatings with different BNNS@AAm contents were prepared and denoted as Films A‐C. Film A was prepared without BNNS@AAm, while Film B and Film C were prepared by introducing 15 mg and 30 mg of BNNS@AAm powder, respectively; all other components (PVA, PVP, AAm, MBAA, APS, and glutaraldehyde) and processing parameters were kept identical. The detailed formulation parameters of Films A‐C, including the BNNS@AAm contents in Films B and C, are summarized in Table S1. For Films B and C, BNNS@AAm powder and PVP were co‐dissolved in an ethanol/water mixture (1:1) and subjected to ultrasonic treatment for 4 h to obtain a homogeneous suspension. For Film A, the same procedure was performed in the absence of BNNS@AAm. The suspension was centrifuged at 4000 rpm for 20 min, and the supernatant was collected to yield a stable dispersion. Separately, polyvinyl alcohol (PVA, 10 wt.%) was soaked at room‐temperature for 30 min and subsequently magnetically stirred at 80°C for 2 h until a transparent solution was obtained. After cooling to room‐temperature, the dispersion was added dropwise into the PVA solution under magnetic stirring at 50°C for 30 min. Subsequently, 5 wt.% AAm monomer and 0.5 wt.% MBAA was introduced, and the mixture was stirred for another 30 min at 50°C. After cooling to room temperature, 0.1 wt.% APS was added as an initiator, and the solution was stirred for 5 min. Finally, 0.5 wt.% glutaraldehyde was added, and the mixture was stirred at 60°C for 15 min to promote crosslinking. The resulting precursor solutions were coated onto clean silicon wafers by spin coating at 1000 rpm to form uniform films. The final coating thicknesses of Films A, B, and C were 142.7, 146.1, and 139.9 µm, respectively, indicating no obvious thickness variation with increasing BNNS@AAm loading (Figure S1). The films were cured by drying at 60°C for 2 h, followed by UV irradiation (365 nm) for 10 min. Residual surface species were removed by rinsing with deionized water, and the coatings were dried under vacuum at 40°C until constant weight was achieved.

### 2.4 Characterizations

Multiple characterization techniques were employed to systematically analyze the structural, chemical composition, thermal, and mechanical properties of the samples. The microstructure and morphology were examined using transmission electron microscopy (TEM, Thermo Fisher Talos F200X, USA) operated at 200 kV and scanning electron microscopy (SEM, ZEISS Sigma 300, Germany) operated at 5 kV after Au sputtering. Atomic force microscopy (AFM, Bruker Dimension ICON, USA) was performed in tapping mode with scan areas of 1 × 1 and 5 × 5 µm^2^, and a non‐contact 3D surface profiler (MicroXAM‐800, KLA, USA) recorded surface profiles over an area of 1 × 1 mm^2^. Crystal structure and phase composition were characterized using X‐ray diffraction (XRD, Rigaku Smart Lab, Japan) with Cu Kα radiation (λ = 1.54 Å) and small‐angle X‐ray scattering (SAXS, Xenocs Xeuss 3.0, France) over a q range of 0.05–1.5 nm^−1^. Surface functional groups and vibrational features were analyzed using Fourier transform infrared spectroscopy (FTIR, Nexus 870, Thermo Nicolet, USA) from 4000 to 400 cm^−1^ at a resolution of 4 cm^−1^ for 32 scans, and Raman spectroscopy (LabRAM HR Evolution, Horiba, France) with a 532 nm laser. X‐ray photoelectron spectroscopy (XPS, PHI5000 VersaProbeIII, USA) was performed using a monochromated Al Kα source. Electron paramagnetic resonance (EPR, Bruker EMXplus‐6/1, Germany) measurements were carried out at room‐temperature. Time‐of‐flight secondary ion mass spectrometry (TOF‐SIMS, PHI nano TOF II, ULVAC‐PHI, Japan) was performed using a Bi_3_^2+^ primary ion source with an energy of 30 keV and an ion current of 2 nA, over a raster area of 100 µm × 100 µm in high mass resolution mode. Thermal properties were measured using a simultaneous thermal analyzer (STA, Rigaku, Japan) from room‐temperature to 600°C at 10°C min^−1^ under N_2_, and thermal conductivity was measured by the transient plane source method on samples of 30 mm × 30 mm using a thermal conductivity analyzer (Hot Disk TPS2500S, Sweden). Optical properties were characterized by an ultraviolet‐visible spectrophotometer (UV–vis, Shimadzu UV‐3600i Plus, Japan) from 200 to 800 nm. Gas barrier properties were measured using a gas permeability tester (BTY‐B2P, Languang Electromechanical, China), and OTR was tested at 23°C and 0% RH. Mechanical properties were tested with an electronic universal testing machine (CMT6103, Mites, China) at a crosshead speed of 50 mm min^−1^. All instruments were operated according to standard operating procedures, with critical experiments repeated to ensure data reliability.

### 2.5 Atomic Oxygen Irradiation

Atomic oxygen (AO) irradiation experiments were carried out at the Lanzhou Institute of Chemical Physics, Chinese Academy of Sciences, using a ground‐based AO simulation facility. The coating samples were exposed to an AO flux of 20 × 10^15^ atoms·cm^−2^·s^−1^ for 0.5 h or 1 h, respectively. After AO irradiation, the samples were collected for subsequent structural characterization, mechanical and friction testing.

### 2.6 Friction Tests

The macro‐friction properties of metal balls and coatings were thoroughly tested using a CSM universal friction tester (TRB3, Switzerland). Test conditions: frequency 0.5 Hz, friction stroke 5 mm, test duration 30 min. The upper specimen used was a 6 mm steel ball, while the lower specimen was a coated silicon wafer measuring 10 mm × 10 mm × 1 mm. The friction atmosphere consisted of oxygen and vacuum conditions. To ensure the reliability of friction results, each test was repeated three times, and the friction tester must undergo zero calibration before each test.

### 2.7 DFT Calculations

The adsorption behaviors of the AAm molecule on the h‐BN monolayers with different defects were investigated by DFT calculations using the Vienna Ab initio Simulation Package (VASP) [47]. The electron−ion interactions of each atom were expressed using a plane wave (PW) basis and the projector‐augmented wave (PAW) pseudopotentials [48]. The generalized gradient approximation (GGA) with Perdew–Burke–Ernzerhof (PBE) functional was employed for the exchange‐correlation potential, and long‐range dispersion interactions were corrected using the DFT‐D3 method [49, 50]. The energy cutoff was set at 500 eV for the wave function expansion. The geometric optimization was achieved by setting the convergence thresholds for energy and atomic force as 1 × 10^−5^ eV and 0.02 eV/Å, respectively. For the h‐BN monolayer, we considered four different conditions, i.e., the pristine structure without any defects, N atom vacancy (V_N_), B atom vacancy (V_B_), and B‐N vacancies (V_BN_). The binding energy between the AAm molecule and the h‐BN monolayer was calculated as *E*_binding_ = *E*_system_—*E*_AAm_—*E*_h‐BN_, where *E*_system_ is the energy of the whole system upon adsorption of the AAm molecule, *E*_AAm_ and *E*_h‐BN_ are the energies of the isolated AAm molecule and the h‐BN monolayer, respectively. The charge density difference distributions between AAm and different h‐BN monolayers were analyzed using the VASPKIT package [51].

### 2.8 Molecular Dynamics Simulations

The role of h‐BN in the irradiation response is further investigated through molecular dynamics simulations performed using the LAMMPS package [52]. To account for the breaking and formation of chemical bonds induced by AO irradiation, the reactive force field (ReaxFF) was employed to describe the interatomic interactions in different systems. This ReaxFF has been successfully applied in simulating boron nitride and the oxidation process [53, 54]. For simplicity, three different cases are considered as one the pure polymer and another two are polymer covered by one or two h‐BN layers (Figure S2). We considered two types of polymer compositions to evaluate their resistance to AO, namely pure PVA and PVA containing 10 wt.% AAm. The bottom region of the polymer, with a thickness of 15 Å, was kept fixed to prevent unintended movement of the entire system. The initial structures of polymer and polymer/h‐BN were first equilibrated at 300 K for 25 ps in the NVT ensemble. Subsequently, the systems were subjected to irradiation by iteratively injecting AO into the matrix, with each AO assigned a velocity of 0.2 Å/fs in the z‐direction toward the h‐BN/PVA. The total number of AO irradiation events for each system was set to 500 with an interval of 5 ps, which is sufficient for the structural response to reach saturation. The atomic snapshots of systems under irradiation were visualized using the OVITO package [55].

## 3 Results and Discussion

**FIGURE 1:**
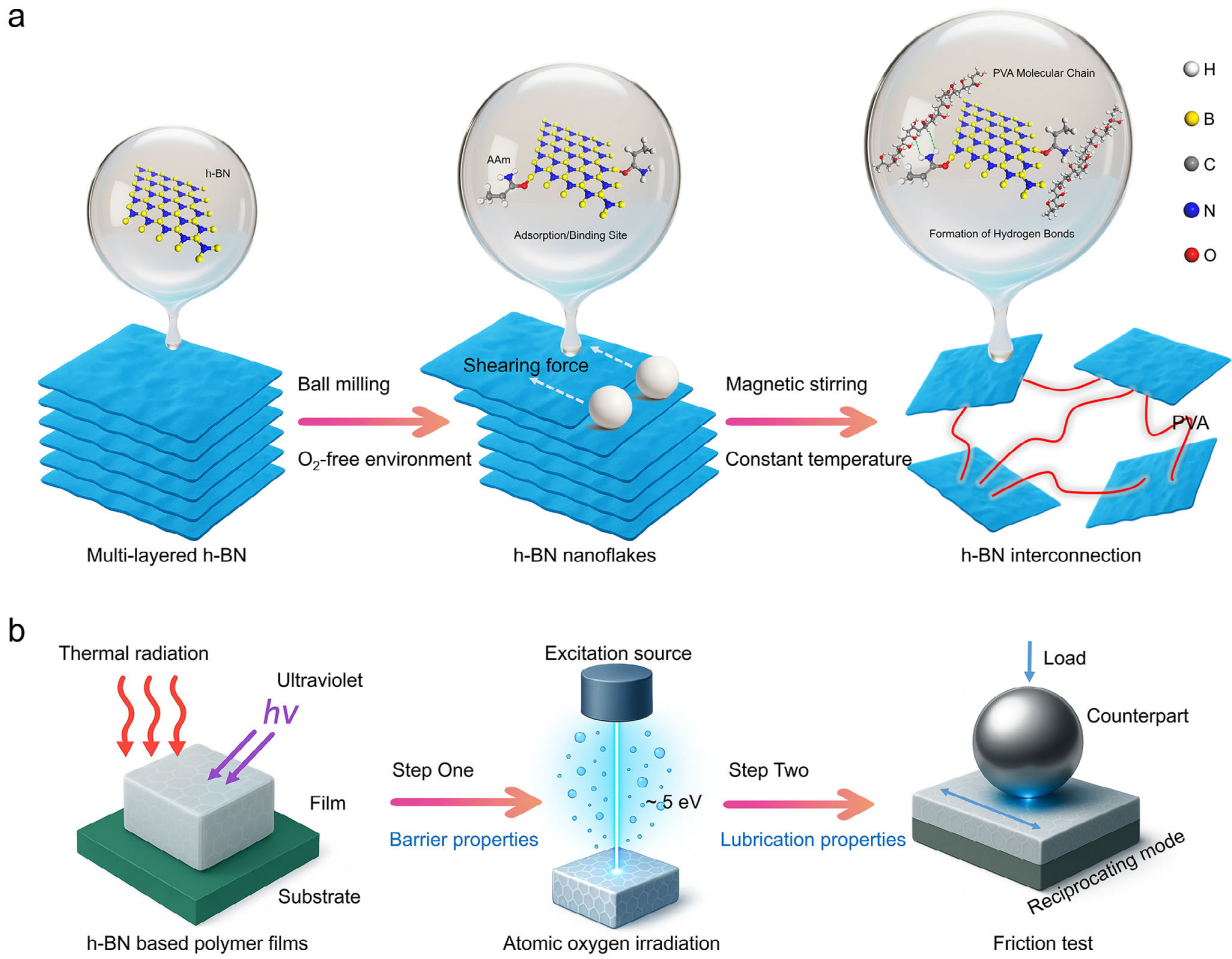
Schematic diagram of the synthesis process and performance testing for the composite coating. (a) Exfoliation of multi‐layered h‐BN into nanoflakes, surface functionalization with AAm, and integration with PVA to form a stable composite network; (b) The coating undergoes thermal radiation and UV exposure for crosslinking, followed by atomic oxygen irradiation and friction testing to evaluate barrier and lubrication properties.

With the assistance of AAm molecules, multilayer h‐BN was exfoliated through ball milling in an oxygen‐free environment (Figure 1a), achieving efficient cleavage of the layered structure. The resulting BNNSs, featuring significantly reduced thickness and enhanced dispersion while preserving their crystalline structure, provide a solid foundation for constructing ordered and stable composite systems. Beyond that, a stable AAm organic modification layer is formed on BNNS as anchors for binding the PVA polymer chain, facilitating the formation of a three‐dimensional BNNS network through hydrogen‐bonding forces. This design strategy leveraged the structural integrity of BNNS and the multifunctional role of AAm, laying the foundation for synergistic improvements in thermal stability, mechanical strength, friction performance, and radiation resistance. Further, the composite system underwent dual curing via thermal and UV crosslinking (Figure 1b), in which UV irradiation initiates crosslinking of the AAm segments, while thermal treatment reinforces intermolecular interactions and improves the intrinsic thermal stability. This dual‐curing method ensures durable bonding between BNNS and the PVA, addressing the typically weak interfacial adhesion in conventional filler‐polymer systems [43]. Finally, the composite coating was evaluated through resistance to AO irradiation and friction testing under the simulated space‐environment condition, where testing conducted at an AO flux of approximately 20 × 10^15^ atoms·cm^−2^·s^−1^ for 0.5 h or 1 h is equivalent to nearly one and a half months or three months of service exposure in low Earth orbit, respectively. This rigorous setup effectively reproduced the destructive effects of high‐energy AO bombardment, providing quantitative validation of the coating's radiation resistance.

**FIGURE 2:**
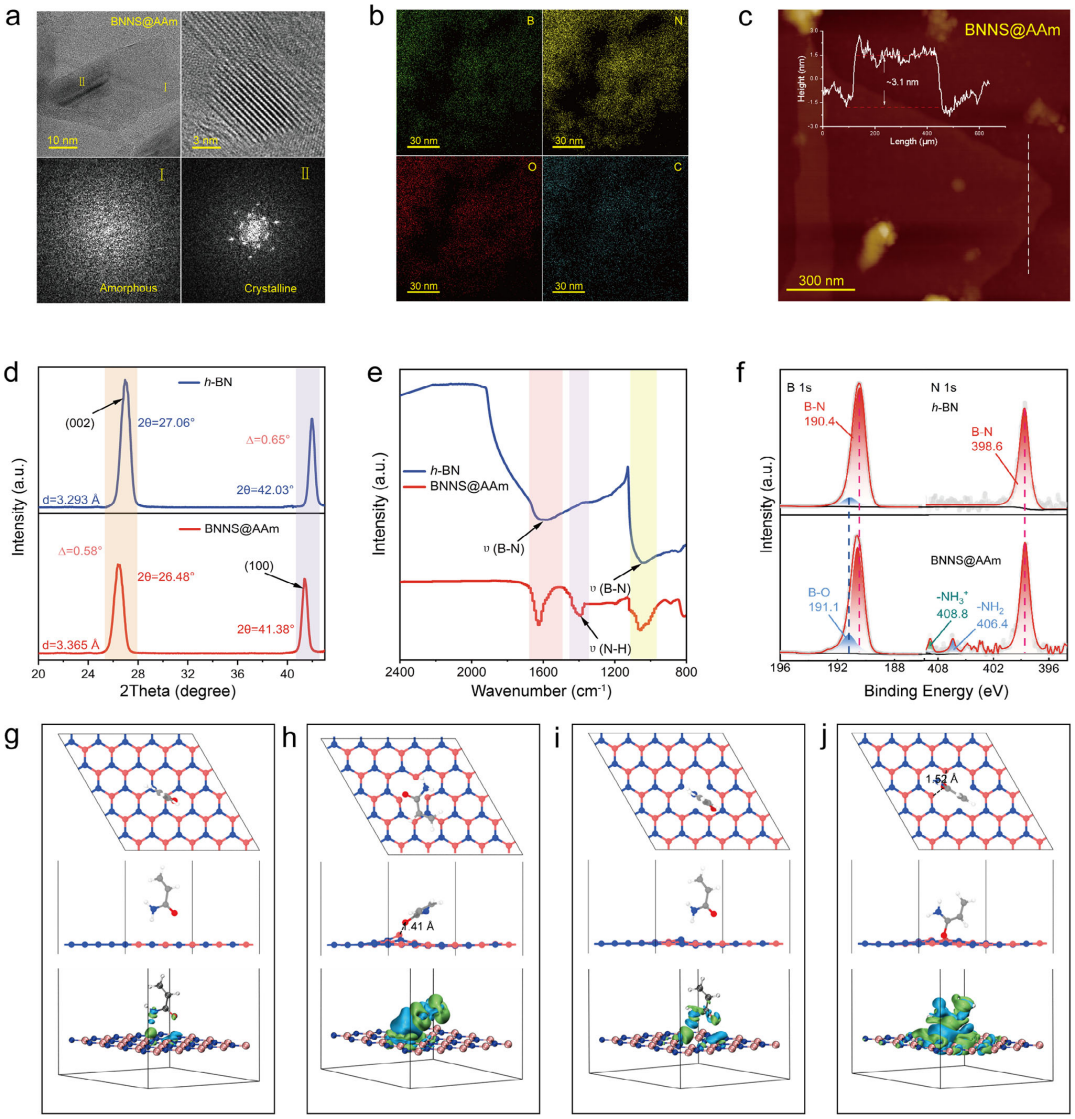
Structural and surface modification characterization of BNNS@AAm. (a) HRTEM image, (b) EDS mapping, and (c) AFM image of BNNS@AAm. (d) XRD spectrum, (e) FTIR spectrum, and (f) XPS B 1s and N 1s spectra of h‐BN and BNNS@AAm. (g–j) DFT calculations of the AAm molecule adsorbed on pristine monolayer h‐BN and h‐BN containing various types of defects: (g) pristine h‐BN, (h) N atom vacancy (V_N_), (i) B atom vacancy (V_B_), and (j) B‐N vacancies (V_BN_). The green and blue clouds represent the electron density difference isosurfaces of 0.001 and ‐0.001 a.u., respectively.

Multidimensional characterizations were first performed to confirm the structural integrity and functionalization of h‐BN. High‐resolution transmission electron microscopy (HRTEM, Figure 2a) revealed that the central domain of BNNS@AAm retained the characteristic long‐range ordered lattice of h‐BN, while disordered stripes appeared at the edges. This stems from a mechanochemical process enabling exfoliation and covalent grafting of AAm functional groups onto the BNNS surface, which collectively disrupt local lattice order. It also indicates that AAm can be uniformly adsorbed on the surface of h‐BN at a minimal loading. This modification prevents nanosheet agglomeration and creates stable organic–inorganic interfacial interactions, which are crucial for forming 3D hydrogen‐bonded networks, as clearly evidenced by elemental mapping (Figure 2b). Additionally, randomly stacked BNNS@AAm with an average thickness of approximately 3.1 nm were detected using atomic force microscopy (Figure 2c), a significant reduction from pristine h‐BN (≈61.3 nm) (Figure S3). AFM‐based statistical analysis further showed that the average lateral diameter decreased from about 5.2 µm for the raw h‐BN to about 3.1 µm for BNNS@AAm, indicating that the exfoliation process was accompanied by a reduction in lateral size (Figure S4). These changes in both thickness and lateral size confirm effective exfoliation and indicate a remarkable increase in the specific surface area and interfacial activity of h‐BN, facilitating compatibility with polymer chains [56].

X‐ray diffraction (XRD, Figure 2d) patterns show that the (002) peak is downshifted from 27.06° for pristine h‐BN to 26.48° for BNNS, corresponding to an interlayer spacing expansion from 3.293 to 3.365 Å, which is due to the introduction of functional groups expanding the interplanar spacing of the h‐BN sheets [57]. After surface functionalization, the peak intensity ratio between I_(100)_ to I_(002)_ is significantly enhanced, indicating that the BNNSs tend to undergo delamination and favorable alignment. These results imply that BNNSs are expected to facilitate polymer chain infiltration and enhance interfacial contact in the subsequent composite coating, thereby achieving self‐dispersion and interfacial load transfer within the polymer matrix [58]. These observations regarding the modification and orientation degree are further supported by Raman spectrum (Figure S5) and Small‐angle X‐ray scattering (SAXS) patterns (Figure S6). The DSC analysis also provides unequivocal proof for the introduction of reactive functional groups onto the chemically inert BNNS surface, where the typical characteristics of free radical polymerization and thermal decomposition processes of AAm molecules occur at 86.6°C and 375.9°C, respectively (Figure S7). Thus, it imparts significant advantages by transforming the inert BNNS into a reactive filler with improved interfacial compatibility, making BNNS@AAm highly suitable for advanced polymer composite coatings.

Figure 2e shows the Fourier Transform Infrared Spectra (FTIR) of h‐BN before and after the AAm molecule modification using ball milling and ultrasonic cavitation methods. The BNNS@AAm displays an N–H bending vibration peak ranging from 1300–1500 cm^−1^, confirming the successful grafting of AAm onto the h‐BN surface. The persistent B–N stretching peak at 1366 cm^−^^1^ demonstrates that the modification cannot alter the h‐BN in‐plane structure, preserving its inherent stability and mechanical properties. X‐ray Photoelectron Spectroscopy (XPS) analysis further elucidates the chemical bonding characteristics and surface compositional profiles of the BNNS@AAm. The high‐resolution B 1s and N 1s spectra (Figure 2f) exhibit a markedly B─O peak intensity and a new N–H peak relative to BNNS@AAm, further confirming the inferences reflected by FTIR. Notably, after modification, the shift in the B 1s peak to 190.4 eV and the unchanged N 1s peak at 398.6 eV indicate that oxygen‐containing and amide functional groups were introduced without altering the core h‐BN structure, preserving the material's structural integrity. The B─O bond at 191.1 eV signifies a covalent interaction between the h‐BN surface and the oxygen‐containing groups of AAm, and the N–H peak at 406.4 eV confirms the hydrogen‐bonding participation, which is anticipated to improve the compatibility of BNNS with polymer matrices and enhance interfacial interactions [59].

To elucidate the adsorption and interfacial interaction mechanisms between AAm molecules and the BNNS surface, density functional theory (DFT) calculations were conducted (Figure 2g–j). The adsorption energy (*E*_ads_) of the AAm molecule on pristine h‐BN is ‐0.28 eV, indicating relatively weak physisorption, as evidenced by the minimal charge‐transfer between AAm and pristine h‐BN upon adsorption (Figure 2g). This behavior largely resembles the previous result of C_2_H_5_OH adsorbed on pristine h‐BN (‐0.27 eV), highlighting the necessity of h‐BN treatment to enhance the binding, e.g., ball milling to introduce defects [60]. To this end, we considered three types of defects on h‐BN monolayers to enhance the reactivity, i.e., N atom vacancy (V_N_), B atom vacancy (V_B_), and B–N vacancies (V_BN_). The simulation results further reveal distinct adsorption behaviors of AAm on h‐BN, arising from the different reactivities of defect sites. Specifically, the AAm molecule preferentially adsorbs at the V_N_ and V_BN_ sites, with adsorption energies (*E*_ads_) of ‐3.07 and ‐4.04 eV, respectively. The short interatomic distances between B atoms in h‐BN and O atoms in AAm at the V_N_ and V_BN_ sites‐1.41 and 1.52 Å, respectively, indicate the formation of B─O bonds, suggesting relatively strong chemisorption accompanied by pronounced charge‐transfer interactions (Figure 2h–j). This not only highlights the strong molecular‐substrate interactions but also suggests the potential formation of stable coordination bonds, which substantially strengthen interfacial adhesion and overall system stability. The constructed composite model (with the basic structural units illustrated in Figure S2) shows excellent agreement with experimental observations, particularly the increase in B─O bonds detected by XPS and the charge density difference analysis. Together, these findings provide robust validation of the proposed interfacial bonding mechanism. The above analyses confirm that BNNS@AAm successfully achieves surface functionalization through AAm modification while preserving the intrinsic structure of h‐BN. This is supported by the observed interlayer expansion and the combined effects of interfacial hydrogen‐bonding and coordination bonding, which enhance the thermal stability of the composite. These structural and interfacial improvements provide a solid foundation for optimizing the composite's performance.

**FIGURE 3:**
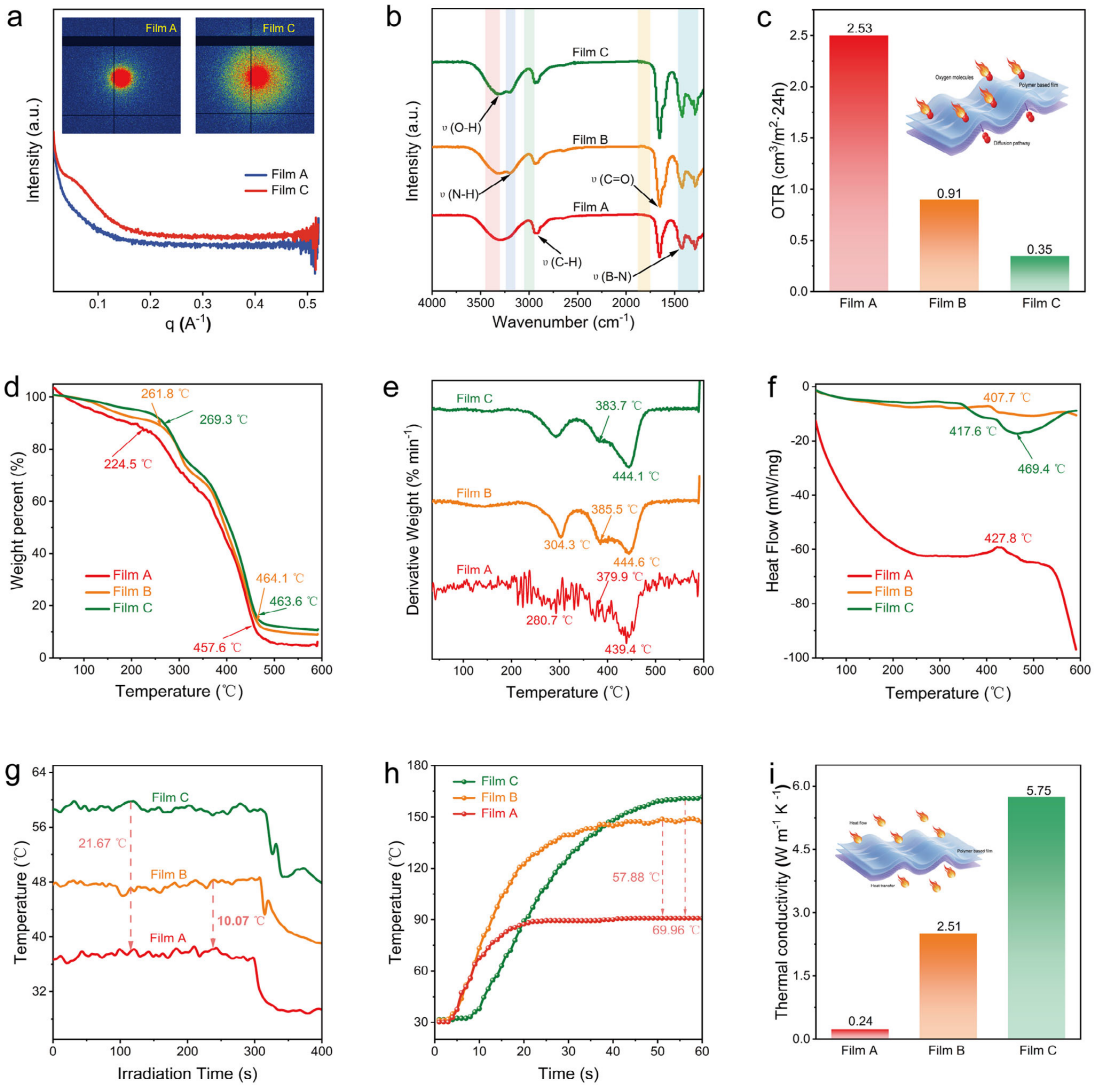
Structural order and thermal properties of BNNS@AAm/PVA composite films. (a) SAXS patterns of Film A and Film C. (b) FTIR spectra, (c) OTR (d) TG curves, (e) DTG curves, (f) DSC curves, (g,h) photothermal response curves, and (i) thermal conductivity of Film A, Film B, and Film C, respectively.

Then, the synthesis and structure of BNNS@AAm based composite films are systematically evaluated. A stable sol suitable of main components, including BNNS@AAm with different contents and PVA, was formed for spin coating conditions. Combined with the subsequent heat and UV treatment, a flexible, dense, and high‐adhesion film was constructed. Cross‐sectional and surface SEM observations (Figure S8) corroborated the structural features, showing that the composite film exhibited a dense and uniform cross‐section morphology with only a few isolated pores, as well as a smooth and compact surface with markedly reduced defects. SAXS analysis (Figure 3a) further reveals that Film C exhibits the sharpest and most intense scattering peak, indicating that uniformly dispersed BNNS@AAm promotes a more ordered layered orientation within the polymer matrix [61, 62]. FTIR (Figure 3b) displayed characteristic ‐NH, ‐OH, and ─C═O absorption bands, confirming the formation of a robust interfacial network between functionalized BNNS and PVA through hydrogen‐bonding and intermolecular interactions.

These structural and interfacial modifications directly translated into improved gas barrier performance. As shown in Figure 3c, the oxygen transmission rate (OTR) decreased from 2.53 cm^3^·m^−^^2^·24 h^−^^1^ for Film A to 0.91 cm^3^·m^−^^2^·24 h^−^^1^ for Film B, and further to 0.35 cm^3^·m^−^^2^·24 h^−^^1^ for Film C, representing nearly an order‐of‐magnitude difference. This significant reduction in OTR is consistent with the synergistic barrier contributions of BNNS@AAm and the strengthened polymer‐filler interface. Specifically, the layered BNNS@AAm creates a more tortuous diffusion pathway for oxygen molecules, thereby increasing the effective transport length. Meanwhile, the interfacial hydrogen‐bonding network between BNNS@AAm and the polymer matrix promotes compact packing and suppresses pore‐ and defect‐associated fast‐transport channels, in accordance with the reduced defects and isolated pores observed in SEM (Figure S8). Together, these effects account for the markedly enhanced gas‐barrier performance of the composite films.

Beyond the enhanced gas barrier properties, the reinforcing effect of BNNS@AAm was further corroborated by thermal stability measurements. TGA and DTG (Figure 3d,e) reveal that Film A began to decompose at 224.5°C, whereas the initial decomposition temperatures of Film B and Film C increased to 261.8°C and 269.3°C, respectively. In addition, Film C exhibits a higher residual mass and a markedly slower decomposition rate in the high‐temperature region. DSC analysis (Figure 3f) shows pronounced exothermic peaks at elevated temperatures. Notably, the peak position of Film C shifts upward by approximately 40°C, from 427.8°C for Film A to 469.4°C, which can be attributed to high‐temperature thermal oxidation and decomposition processes rather than glass transition. These results indicate that BNNS@AAm effectively suppresses the disordered motion of polymer segments through interfacial interactions, enhances molecular chain confinement, and thereby significantly improves the thermal stability and high‐temperature structural integrity of the film [63, 64]. This enhanced thermal stability is particularly important under the coupled AO irradiation and frictional heating conditions encountered in LEO, where delayed thermal degradation helps to maintain coating integrity over prolonged service times.

The improvement in thermal conductivity is particularly noteworthy. As shown in Figure 3i, Film A exhibits a thermal conductivity of only 0.24 W·m^−^^1^·K^−^^1^, whereas Film B and Film C reach the values of 2.51 and 5.75 W·m^−^^1^·K^−^^1^, respectively, with Film C showing a nearly 24‐fold enhancement compared to the pristine film. To further clarify the role of interfacial modification, an untreated‐BN sample was also examined under otherwise identical conditions. Its thermal conductivity is only 1.32 W·m^−1^·K^−1^ (Figure S9), indicating that the enhanced thermal transport in the present system is related not only to BN incorporation, but also to the AAm‐assisted interfacial modification. This dramatic improvement results from the uniform dispersion and oriented alignment of BNNS@AAm within the polymer matrix, which establishes continuous phonon transport pathways. Additionally, interfacial hydrogen‐bonding between BNNS@AAm and PVA chains reduces thermal resistance at the interface, facilitating more efficient heat transfer across the composite. These findings align with previous reports on h‐BN/polymer composites, further confirming that thermal conductivity depends on both filler dispersion and interfacial bonding, with functionalization serving as the key to synergizing these two factors [65, 66].

Photothermal and thermal response further verified the thermal management potential of the composite films. In the photothermal measurements (Figure 3g), the steady‐state temperature of Film B is 10.07°C higher than that of Film A, while Film C exceeds Film A by 21.67°C, indicating that the ordered orientation network of BNNS@AAm significantly enhances light‐heat‐phonon coupling, thereby enabling faster heating and higher steady‐state temperatures. The thermal response tests (Figure 3h) reveal even more pronounced improvements, with the steady‐state temperatures of Film B and Film C being 57.88°C and 69.96°C higher than that of Film A, respectively, demonstrating that BNNS@AAm enhances both heat conduction and energy transfer efficiency and thus leads to stronger thermal responsiveness under heating conditions. Infrared thermal imaging during the cooling stage (Figure S10) provided additional evidence, showing transient surface temperatures of 62.6°C, 52.1°C, and 44.0°C for Films A, B, and C, respectively, with Film C exhibiting the lowest surface temperature and the most uniform temperature distribution. Taken together, the photothermal, thermal response, and infrared thermal imaging results demonstrate that BNNS@AAm‐functionalized films exhibit enhanced photothermal conversion and thermal responsiveness, along with improved heat dissipation and temperature uniformity, thereby achieving synergistic optimization of heating, steady‐state temperature control, and cooling behavior.

Furthermore, the potential of the composite film for thermal radiation dissipation was validated by infrared emissivity measurements (Figure S11). Film A exhibited an emissivity of approximately 0.923, whereas Film C reached 0.986, maintaining consistently high values across the 2.5–15 µm infrared wavelength range. When combined with the enhanced thermal conductivity and improved temperature uniformity, the elevated emissivity demonstrates that the incorporation of BNNS@AAm markedly strengthens the radiative heat dissipation capability of the films, thereby enabling more efficient and integrated thermal management [67]. This picture is fully consistent with the previously discussed thermal conductivity and infrared thermal imaging results and highlights the superior heat dissipation and temperature regulation capacity of Film C under extreme conditions.

Therefore, the incorporation of BNNS@AAm markedly enhances the structural order, gas‐barrier capability, and thermal management of the composite films, with Film C consistently outperforming Films A and B across all evaluated metrics. This multidimensional improvement highlights the pivotal role of BNNS@AAm functionalization and controlled dispersion in constructing continuous transport pathways and defect‐suppressed interfaces. Taken together, these findings demonstrate that simultaneous optimization at both the structural and interfacial levels is crucial for achieving synergistic property enhancement and provide a solid basis for the subsequent analysis of the coating's mechanical retention capability and tribological stability under AO irradiation.

**FIGURE 4:**
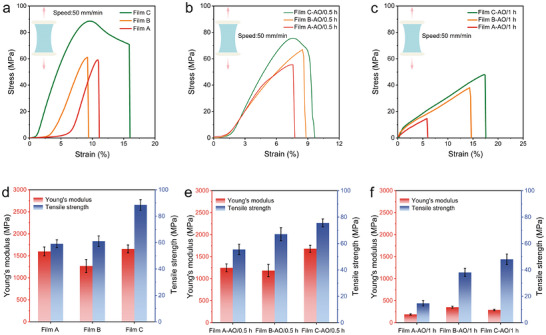
Mechanical properties of BNNS@AAm/PVA composite films. (a) Stress–strain curves before irradiation; (b) Stress–strain curves after 0.5 h of AO irradiation; and (c) Stress–strain curves after 1 h of AO irradiation. (d–f) Comparison of Young's modulus and tensile strength at different AO irradiation durations.

Having established the advantages of the composite films in terms of structural order and thermal properties, their mechanical performance and retention capability under atomic oxygen environments were evaluated. Figure 4a presents the stress–strain behavior of the three films under non‐irradiated conditions. Film A (without h‐BN) exhibited low strength (∼59.2 MPa) and modulus (∼1599 MPa), providing limited load‐bearing capacity. With the introduction of BNNS@AAm, Film B shows a slight increase in strength (∼61.1 MPa), but its modulus decreased to ∼1267 MPa, suggesting that low filler content improves strength without enhancing stiffness. In contrast, Film C exhibits the highest strength (∼88.5 MPa) and modulus (∼1656 MPa), demonstrating that the uniform dispersion and strong interfacial interactions of high‐content BNNS@AAm significantly enhanced the combined strength and stiffness of the composite film. This trend is further confirmed in the bar chart of Figure 4d, where Film C clearly outperforms the other two films, validating the effectiveness of filler incorporation in mechanical reinforcement.

After 0.5 h of AO irradiation (Figure 4b,e), all three films exhibited noticeable mechanical degradation, though the extent varied. Film A showed a rapid decline, with strength and modulus reduced to ∼55.5 and ∼1248 MPa, respectively, indicating severe erosion of polymer segments under AO attack. Film B experienced a further decrease in modulus (∼1184 MPa), while its strength slightly increased to ∼67.1 MPa, suggesting that limited crosslinking or chain rearrangement may have temporarily enhanced toughness. In contrast, Film C maintained the most favorable properties, with a strength of ∼75.6 MPa and modulus of ∼1681 MPa, demonstrating its ability to preserve mechanical stability during early irradiation. Such contrasting degradation behavior under early‐stage AO exposure highlights that the layered barrier effect and interfacial hydrogen‐bond constraints introduced by BNNS@AAm effectively mitigate AO‐induced chain scission, thereby slowing the deterioration of mechanical properties.

When irradiation time is extended to 1 h (Figure 4c,f), the mechanical properties of all three films further deteriorate. Film A's strength plummeted to ∼14.7 MPa, with a modulus of only ∼190 MPa, nearly losing its load‐bearing capacity; Film B maintained the highest modulus (∼353 MPa) but exhibited brittle behavior, with strength dropping to ∼38.1 MPa. In contrast, Film C retained a modulus of ∼290 MPa and strength of ∼48.1 MPa, significantly lower than the non‐irradiated state but still superior to the control group. After 1 h of AO irradiation, Film C still preserved ∼54% of its initial tensile strength and ∼18% of its modulus, whereas Film A retained only ∼25% and ∼12%, respectively, confirming that Film C exhibits the most favorable residual mechanical performance. This pronounced contrast demonstrates that the high intrinsic stability of BNNS@AAm, together with its strong interfacial interactions with the polymer matrix, is crucial for retaining the mechanical integrity of the composite films under atomic oxygen irradiation.

**FIGURE 5:**
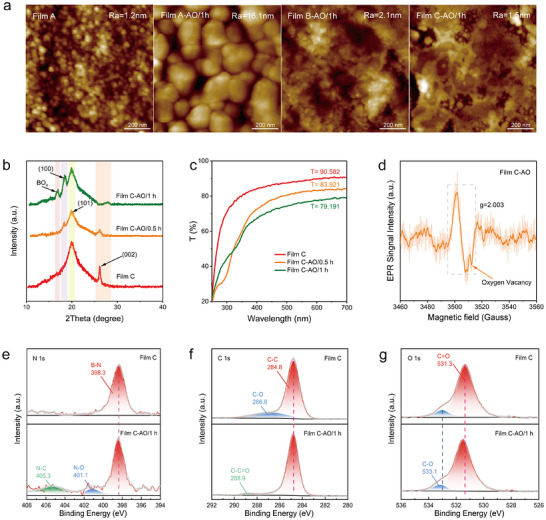
Structural and chemical evolution under AO irradiation. (a) AFM images of Film A and Films A, B, and C after 1 h of AO irradiation. (b) XRD spectra and (c) UV transmittance of Film C before irradiation, after 0.5 h of AO irradiation, and after 1 h of AO irradiation. (d) EPR spectrum of Film C after AO irradiation. (e–g) XPS N 1s, C 1s, and O 1s spectra of Film C and Film C after 1 h of AO irradiation.

To elucidate the structural evolution and chemical mechanisms of films under atomic oxygen (AO) irradiation, systematic multiscale characterization was conducted. AFM surface topography (Figure 5a) reveals that non‐irradiated Film A exhibits a smooth surface with a roughness (Ra) of approximately 1.2 nm. After 1 h of AO irradiation, the surface roughness increases sharply to 16.1 nm, showing distinct agglomeration and pitting, indicating severe degradation of the pure polymer. In contrast, Films B and C retain relatively uniform surfaces, with roughness values of 2.1 and 1.6 nm, respectively, under identical conditions. This demonstrates that the incorporation of BNNS@AAm significantly suppressed AO‐induced erosion, conferring superior interfacial stability and structural integrity. Large‐scale AFM images (Figure S12) further corroborated this inference, showing that Film A's roughness increased to 19.3 nm after 1 h AO exposure, with extensive granular damage, while Films B and C maintained roughness values of 2.9 and 1.9 nm, respectively, with intact, continuous surfaces. These observations confirm that the layered barrier effect of BNNS@AAm effectively prevents AO‐induced surface roughening and micro/nano‐scale degradation, reflecting the physical barrier role of BNNS in blocking direct AO impact on the PVA substrate.

XRD analysis (Figure 5b) provides further insight into the crystalline evolution. The non‐irradiated Film C predominantly displayed typical h‐BN diffraction peaks, including the (002) and (101) planes, indicative of a well‐stacked layered structure with in‐plane order. After 0.5 h of AO irradiation, the (002) peak intensity markedly decreased, and a new (100) peak appeared, suggesting disruption of interlayer order and the emergence of in‐plane periodicity [68]. Extending irradiation to 1 h further amplified this trend: the (002) peak continued to weaken, the (100) peak persisted, and a new B‐O_x_ peak emerged in the low‐angle region, indicating oxidation side reactions at defect and edge sites. This diffraction evolution under AO exposure reveals a synergistic structural response, characterized by weakened interlayer order, enhanced in‐plane features, and mild oxidation at defect and edge sites, while the overall h‐BN framework remains structurally intact.

Optical transmittance measurements (Figure 5c) further corroborate the limited extent of AO‐induced degradation. Film C exhibits ∼90% transmittance in the visible range, which gradually decreases to ∼79% after prolonged AO irradiation. Despite this reduction, the transmittance remains higher than that of many inorganic composites, indicating that AO erosion does not cause severe optical or structural damage and that the coating maintains robust resistance to irradiation‐induced corrosion [69]. EPR spectroscopy (Figure 5d) provides additional evidence of irradiation‐induced defects. After AO exposure, Film C exhibits a pronounced g ≈ 2.003 signal, attributed to oxygen vacancy formation. This defect‐induced restructuring of the chemical environment is consistent with the attenuation of the (002) peak and the emergence of the B‐O_x_ peak observed in XRD.

XPS analysis provides direct insight into the interfacial chemical evolution (Figure 5e–g). In the N 1s spectrum, AO irradiation introduces new N─C and N─O peaks alongside the original B‐N signal, indicating that nitrogen atoms participate in both interactions with polymer chains and oxidation reactions. In the C 1s spectrum, the original C–O peak disappears and is replaced by an O─C═O peak, signifying a transition from hydroxyl/ether configurations to carboxyl‐like structures. Concurrently, the O 1s signal intensity increases, consistent with the formation of additional oxygen‐containing species. Taken together, the N 1s, C 1s, and O 1s spectra reveal that AO irradiation promotes deep interfacial reactions between BNNS and the polymer chains, driving reconstruction of the local bonding environment. Raman spectroscopy (Figure S13) further supports this interfacial reconstruction scenario. The non‐irradiated Film C exhibits the characteristic E_2g_ vibrational peak of h‐BN at 1366 cm^−1^, along with O–H vibrational signals from the polymer matrix. After irradiation, the intensity of the E_2g_ peak decreases, while the O–H signal intensifies, indicating that AO preferentially attacks edges and functional group sites, weakening interlayer coupling within BNNS and introducing additional hydroxyl groups [70]. The Raman observations are consistent with the functional group rearrangements observed in XPS, confirming irradiation‐induced interfacial chemical reconstruction. This reconstruction, in which BNNS actively participates in oxidation and bonding rearrangements with PVA chains, creates a chemically robust interphase that enhances AO resistance while preserving optical transparency.

**FIGURE 6:**
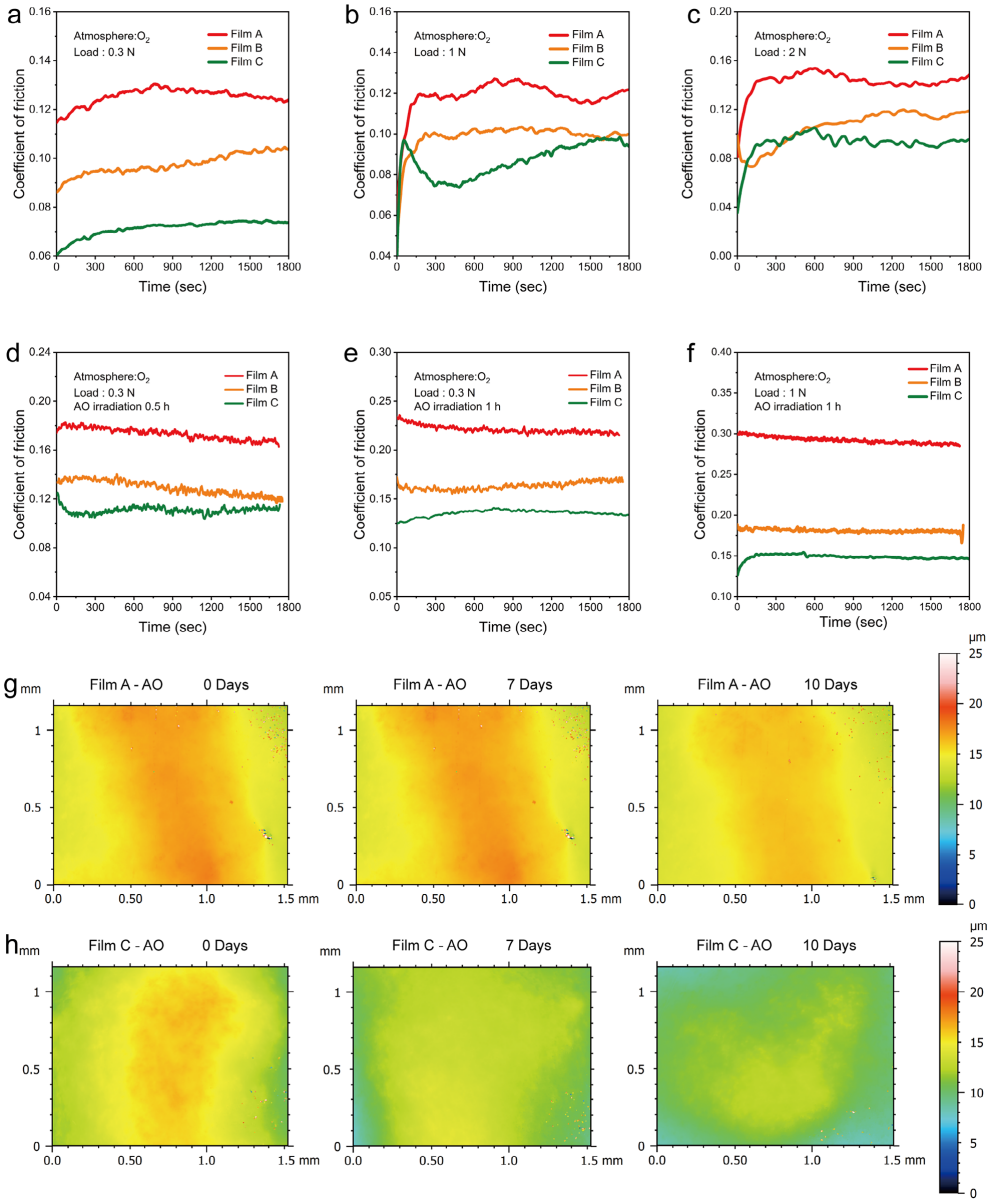
Tribological performance under different loads and AO irradiation conditions. Friction curves of Film A, Film B, and Film C in an oxygen environment (a‐c) without irradiation or (d–f) after AO irradiation, respectively. (g,h) 3D profiles of the wear scar of Film A and Film C at different time points after AO irradiation.

The friction tests were used to reveal their wear resistance and radiation‐induced retention capability under varying loads and AO irradiation conditions. Under non‐irradiated conditions (Figure 6a–c), the friction behavior of the three films exhibits distinct differences with increasing load. Film A maintains a friction coefficient (CoF) of 0.12–0.14 at low loads (0.3 N), gradually increasing and exhibiting high and unstable friction. Film B shows a slight improvement, with a CoF of approximately 0.10, though it still exhibits fluctuations. In contrast, Film C consistently maintains a low CoF of 0.07–0.08, even under higher loads of 1 N and 2 N, where the CoF remains stable at 0.09–0.10. Such distinct friction responses, particularly the low and stable CoF of Film C over a wide load range, highlight that uniformly dispersed and well‐oriented BNNS@AAm constructs continuous solid lubrication pathways within the matrix, enabling efficient load distribution and markedly reducing friction while maintaining interfacial stability.

After 0.5 h of AO irradiation (Figure 6d), the CoF of all three films increased, yet significant differences remained. The CoF of Film A rises to ∼0.20 and continues to climb, indicating rapid surface degradation. Film B stabilizes around ∼0.14, with some fluctuation, while Film C remains stable at ∼0.09–0.10, showing minimal change. This indicates that the barrier effect of BNNS@AAm effectively suppressed the direct erosion of polymer chains by AO, maintaining stable interfacial lubrication. When the irradiation time is extended to 1 h (Figure 6e,f), the friction properties further deteriorate. Under a 0.3 N load, the CoF of Film A increases to ∼0.25 and approaches 0.30 at 1 N, indicating severe degradation. Film B's values are ∼0.16 and ∼0.19, showing modest improvement over Film A but still exhibiting evident wear. In contrast, Film C preserves CoF of ∼0.12 and ∼0.14 under identical conditions, maintaining stable curves and highlighting its exceptional wear resistance and radiation tolerance. A literature‐based comparative plot (Figure S14) further shows that the present coating remains in a relatively low‐friction regime both before and after AO irradiation compared with representative systems, highlighting its competitive tribological stability under AO exposure.

The evolution of the wear‐track morphology was tracked using a three‐dimensional surface profiler (Figure 6g,h). Film A exhibits a wear scar depth approaching 25 µm after AO irradiation, with minimal recovery observed after 7 and 10 days of static storage, indicating irreversible surface defect expansion. In contrast, Film C exhibits an initial scratch depth below 15 µm, which gradually diminishes over time. After 10 days, the surface tended toward flatness, with noticeable contraction of the scratched area. This suggests that BNNS@AAm not only reduces wear during friction but also imparts surface self‐healing capability under environmental stress. Combined with stable friction performance, the layered barrier and interfacial chemistry of BNNS@AAm enhance anti‐AO properties while facilitating dynamic surface reconstruction, improving tribological performance and radiation resistance. This significantly extends the composite film's service life in extreme space environments.

The radar chart (Figure S15) provides a multidimensional comparison of the three films across six representative performance parameters. The revised presentation more directly highlights the overall multifunctional advantage of Film C. The schematic of the tribological experiment (Figure S16) illustrates the steel ball/film contact state and load transfer pathway during friction testing, providing methodological support for the experimental findings. Collectively, these results demonstrate that Film C consistently maintains low CoF, exceptional wear resistance, high stability and radiation tolerance under both conventional and AO‐irradiated conditions.

**FIGURE 7:**
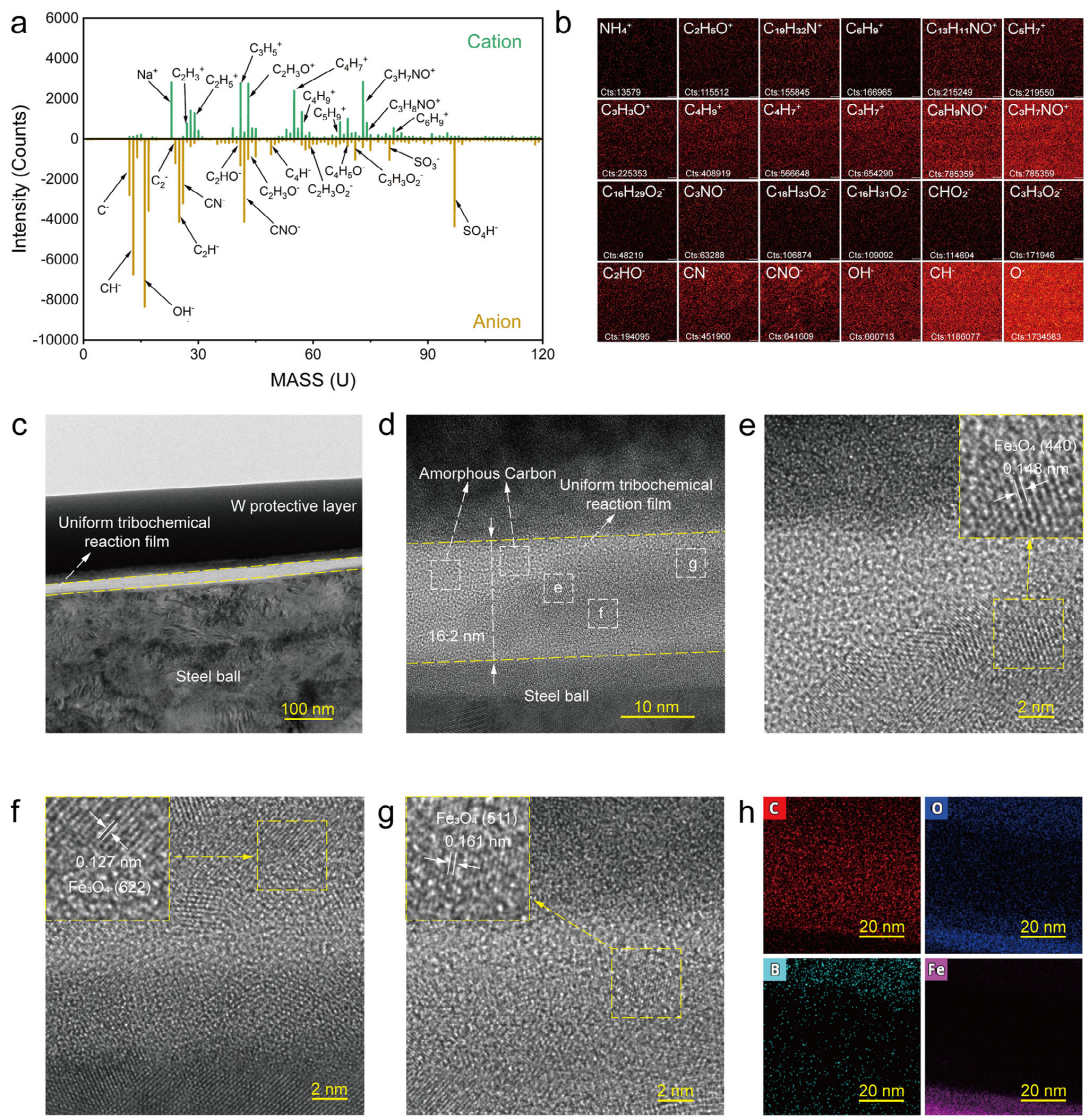
Structural and Compositional Characterization of the Transfer Film After Friction. (a,b) TOF‐SIMS mass spectra of cations and anions generated from wear tracks and corresponding 2D TOF‐SIMS images. (c,d) TEM images of cross‐sections of steel ball wear tracks. (e–g) HRTEM images of different locations within the wear tracks (insets show magnified regions). (h) corresponding EDS mapping analysis for C, O, B, and Fe of the tribofilm on the steel ball.

Motivated by the distinct frictional stability and wear evolution of the composite films, we next probed the underlying anti‐AO and wear‐resistant mechanisms by performing detailed multiscale characterizations of the transfer film formed on the friction pair surface. TOF‐SIMS mass spectrometry (Figure 7a) detected abundant fragment ions such as C_x_H_y_^+^, C_x_H_y_O^+^, CN^−^, and CNO^−^ within the wear scar region, indicating that polymer segments underwent chain scission, oxidation, and recombination under the coupled effects of AO irradiation and friction. SIMS imaging (Figure 7b) revealed uniform enrichment of ions like CxHy^−^and CN^−^across the surface, rather than localized distributions, demonstrating that surface chemical reactions reorganized the interfacial environment macroscopically. These findings align with XPS results (Figure 5e–g) showing the disappearance of C‐O bonds, the emergence of O─C═O peaks, and the appearance of N─C and N─O bonds, confirming that AO irradiation and frictional shear synergistically induced oxidation and nitridation processes at the interface. Importantly, BNNS‐related ionic signals were also detected, suggesting that BNNS nanosheets were integrated into the tribofilm, along with polymer fragments and oxides, forming a composite protective layer.

Cross‐sectional TEM images (Figure 7c) reveal a uniform, continuous friction‐induced chemical film approximately 15–20 nm thick formed on the steel ball surface, overlaid by a protective deposition layer. High‐resolution TEM (Figure 7d) indicates the film's bulk structure is amorphous, exhibiting structural relaxation under friction and irradiation. Local magnified images (Figure 7e–g) further reveal a small number of nanocrystals embedded within the film. The corresponding interplanar spacings of approximately 0.161, 0.148, and 0.127 nm correspond to the (511), (440), and (533) crystal planes of Fe_3_O_4_, respectively. This aligns with previous reports indicating Fe‐based surfaces readily oxidize to Fe_3_O_4_ during friction, suggesting that AO irradiation and frictional heat jointly induce mild oxidation on the steel ball surface, forming a small number of nanocrystals embedded within the transfer film [71]. These nanocrystals synergize with the amorphous matrix, significantly enhancing the film's structural stability and wear resistance. Elemental distribution maps (Figure 7h) further confirmed the uniform distribution of C, O, B, and Fe within the transferred film. The presence of B indicates that BNNS fragments actively participated in tribofilm formation, with BNNS layers embedded as a rigid framework. Together with polymer fragments and iron oxides, this hybrid structure significantly improves the mechanical stability and oxidation resistance of the interfacial film.

These multiscale results demonstrate that the transfer film formed under the synergistic action of AO irradiation and friction consists of an amorphous organic matrix interspersed with BNNS fragments and a small amount of Fe_3_O_4_ nanocrystals. This multi‐component, multi‐phase architecture provides both chemical passivation and structural reinforcement, thereby endowing the interface with superior wear resistance and long‐term stability in extreme space environments.

**FIGURE 8:**
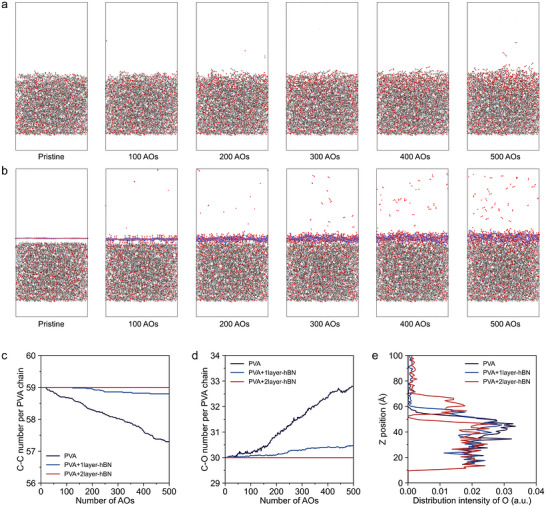
Atomic snapshots of (a) PVA and (b) PVA with mono‐layer h‐BN under sustained AO exposure. Number of (c) C─C and (d) C─O bonds as a function of AOs upon AO erosion. (e) The distribution of O for different systems after 500 AOs exposure.

The atomistic mechanism of AO erosion was further investigated through reactive MD simulations to elucidate the role of h‐BN in enhancing anti‐AO performance. Figure 8a,b present the simulation snapshots of PVA without and with a single h‐BN layer under sustained AO exposure, respectively (Figure S17, PVA with two h‐BN layers). For the pure PVA system, AOs can easily penetrate into the polymer matrix. In contrast, a portion of the AOs bind to the h‐BN layer, leading to partial surface oxidation, while others are reflected due to the shielding effect provided by h‐BN. The evolution of the C─C bond number, which reflects the structural integrity of PVA, indicates that the carbon backbone is continuously degraded under AO irradiation. However, even a single h‐BN layer can significantly mitigate this deterioration, and two h‐BN layers can effectively prevent damage to the PVA matrix (Figure 8c). Meanwhile, Figure 8d shows that the PVA chains are readily oxidized upon AO exposure, whereas the presence of h‐BN effectively mitigates or even prevents oxidation, thereby providing strong protection. Combined with the spatial distribution of oxygen (from both intrinsic and external sources, as shown in Figure 8e and Figure S18), it can be observed that sustained AO exposure leads to the expansion of PVA, while the external AOs are effectively blocked at the surface by the protective h‐BN layer. Besides PVA, we further investigated a more complex system, namely PVA containing 10 wt.% AAm, to evaluate the capability of h‐BN in enhancing AO resistance. The addition of AAm to the PVA matrix enhances the structural integrity of the polymer network by enabling the formation of a three‐dimensional hydrogen‐bonding network between the hydroxyl groups of PVA and the amide groups of AAm, resulting in strong –OH···O═C and –NH···O– interactions (Figure S19). As shown in Figure S20, AO species can still readily penetrate into the polymer matrix in the pure PVA–AAm system, similar to that observed for pure PVA. Similarly, when an h‐BN layer is introduced, part of the AO species interacts with and binds to the h‐BN surface, leading to partial surface oxidation, while the remaining AO species are reflected due to the shielding effect of the h‐BN layer. The introduction of even a single h‐BN layer significantly suppresses the structural deterioration of the polymer matrix, while two h‐BN layers effectively prevent damage to the PVA matrix (Figure S21). These results reveal that AO irradiation deteriorates the PVA chains through both ballistic and oxidative effects, whereas h‐BN prevents AO penetration via self‐oxidation, thereby elucidating the atomistic origin of the AO resistance in the h‐BN/PVA system.

## 4 Conclusions

In conclusion, this study successfully developed BNNS@AAm/PVA composite coatings with enhanced thermal conductivity, mechanical performance, and resistance to AO irradiation. The integration of functionalized BNNS into the PVA matrix significantly improved the material's thermal conductivity, with the thermal conductivity of Film C reaching 5.75 W·m^−1^·K^−1^, nearly 24 times that of pure PVA (0.24 W·m^−1^·K^−1^). Additionally, the advanced composite films exhibited a friction coefficient of 0.12–0.14, tensile strength of 48.1 MPa, demonstrating excellent wear resistance, mechanical stability, and enhanced strength under AO exposure. The OTR of films decreased significantly from 2.53 to 0.35 cm^3^·m^−2^·24 h^−1^, reflecting an enhanced gas barrier performance. Multiple characterizations reveal that defect‐engineered BNNS provides high‐affinity sites for AAm chemisorption and participates in controlled interfacial oxidation and bonding rearrangement with PVA chains, forming a chemically robust interphase that stabilizes the polymer under irradiation. The DFT calculations and MD simulations further confirmed that BNNS@AAm improves interfacial bonding and provides effective AO shielding, ensuring long‐term durability under harsh space conditions. Reactive MD simulations demonstrate that h‐BN layers act as sacrificial AO shields, localizing oxidation within a thin oxidized h‐BN region and suppressing deep chain scission in the underlying PVA. Together with multiscale tribofilm characterizations, it can be established that a hierarchical protection mechanism in which a BNNS‐reinforced barrier delays AO ingress, nanoscale BNNS layers dissipate heat and block AO, and a BNNS‐containing tribofilm passivates and strengthens the sliding interface. This BNNS@AAm‐bridged structural design thus provides a general strategy for lightweight polymer coatings that must simultaneously manage heat, resist AO erosion, and sustain durable low‐friction performance in extreme space environments.

## Conflicts of Interest

The authors declare no conflicts of interest.

## Supporting information




**Supporting File**: advs76093‐sup‐0001‐SuppMat.docx.

## Data Availability

The data that support the findings of this study are available from the corresponding author upon reasonable request.

## References

[1] J.‐H. Han , S.‐H. Seok , Y. H. Jin , et al., “Robust 2D Layered MXene Matrix–Boron Carbide Hybrid Films for Neutron Radiation Shielding,” Nature Communications 14 (2023): 6957, 10.1038/s41467-023-42670-z. PMC10618517 37907547

[2] G. Liu , L. Cheng , X. Luan , and J. Zhang , “Damage Behavior of Atomic Oxygen on CVD SiC Coating‐Modified Carbon/Carbon Composite in Low Earth Orbit Environment,” Journal of Materials Science & Technology 35 (2019): 2957–2965, 10.1016/j.jmst.2019.08.011.

[3] P. Serles , E. Nicholson , J. Tam , et al., “High Performance Space Lubrication of MoS_2_ With Tantalum,” Advanced Functional Materials 32 (2022): 2110429, 10.1002/adfm.202110429.

[4] D. Beck , J. Bickus , E. Klein , et al., “Additive Manufacturing of Multimaterial Composites for Radiation Shielding and Thermal Management,” ACS Applied Materials & Interfaces 15 (2023): 35400–35410, 10.1021/acsami.2c22478. 37289198

[5] B. E. A. Holmes , V. T. A. Oiko , and P. C. E. Roberts , “A Review of Satellite‐Based Atomic Oxygen Sensing Methods,” Progress in Aerospace Sciences 137 (2023): 100886, 10.1016/j.paerosci.2023.100886.

[6] J. Yan , Y. Rong , Y. Zhang , et al., “Wear and Corrosion Resistance of Textured and Functionally Particle‐Enhanced Multilayer Dual‐Biomimetic Polymer Coatings,” Tribology International 202 (2025): 110335, 10.1016/j.triboint.2024.110335.

[7] S. Mao , D. Zhang , Y. Zhang , J. Yang , and J. Zheng , “A Universal Coating Strategy for Controllable Functionalized Polymer Surfaces,” Advanced Functional Materials 30 (2020): 2004633, 10.1002/adfm.202004633.

[8] Y. Gong , G. Li , Y. Pan , et al., “Atomic Oxygen Erosion Mechanism of Polyimide via Reactive Molecular Dynamics Simulation and Density Functional Theory Calculation,” Polymer Degradation and Stability 228 (2024): 110928, 10.1016/j.polymdegradstab.2024.110928.

[9] I. Gouzman , E. Grossman , R. Verker , N. Atar , A. Bolker , and N. Eliaz , “Advances in Polyimide‐Based Materials for Space Applications,” Advanced Materials 31 (2019): 1807738, 10.1002/adma.201807738. 30803081

[10] S. Zhou , L. Zhang , L. Zou , B. I. Ayubi , and Y. Wang , “Deterioration Mechanism of Fluorinated Colorless Polyimide and Polysilazane Reinforcement Under Atomic Oxygen Erosion,” Progress in Organic Coatings 200 (2025): 108962, 10.1016/j.porgcoat.2024.108962.

[11] R. Xu , Y. Zhang , S. Ma , et al., “A Universal Strategy for Growing a Tenacious Hydrogel Coating From a Sticky Initiation Layer,” Advanced Materials 34 (2022): 2108889, 10.1002/adma.202108889. 35014101

[12] X.‐F. Pan , B. Wu , H.‐L. Gao , et al., “Double‐Layer Nacre‐Inspired Polyimide‐Mica Nanocomposite Films With Excellent Mechanical Stability for LEO Environmental Conditions,” Advanced Materials 34 (2022): 2105299, 10.1002/adma.202105299. 34802169

[13] Y. Su , V. G. Kravets , S. L. Wong , J. Waters , A. K. Geim , and R. R. Nair , “Impermeable Barrier Films and Protective Coatings Based on Reduced Graphene Oxide,” Nature Communications 5 (2014): 4843, 10.1038/ncomms5843. 25208890

[14] K. Liang , E. M. Spiesz , D. T. Schmieden , A.‐W. Xu , A. S. Meyer , and M.‐E. Aubin‐Tam , “Bioproduced Polymers Self‐Assemble With Graphene Oxide Into Nanocomposite Films With Enhanced Mechanical Performance,” ACS Nano 14 (2020): 14731–14739, 10.1021/acsnano.0c00913. 33146012 PMC7690046

[15] Y. He , A. Suliga , A. Brinkmeyer , M. Schenk , and I. Hamerton , “Effect of Atomic Oxygen Exposure on Polybenzoxazine/POSS Nanocomposites for Space Applications,” Composites Part A: Applied Science and Manufacturing 177 (2024): 107898, 10.1016/j.compositesa.2023.107898.

[16] D. Wei , F. Zeng , and J. Cui , “Damage and Degradation of Mechanical Properties of Polyimide‐Based Materials Under Atomic Oxygen Attack: A Molecular Dynamics Simulation Study,” Computational Materials Science 243 (2024): 113110, 10.1016/j.commatsci.2024.113110.

[17] C. Lee , X. Wei , J. W. Kysar , and J. Hone , “Measurement of the Elastic Properties and Intrinsic Strength of Monolayer Graphene,” Science 321 (2008): 385–388, 10.1126/science.1157996. 18635798

[18] M. Chhowalla and G. A. J. Amaratunga , “Thin Films of Fullerene‐Like MoS_2_ Nanoparticles With Ultra‐Low Friction and Wear,” Nature 407 (2000): 164–167, 10.1038/35025020. 11001049

[19] S. Bertolazzi , J. Brivio , and A. Kis , “Stretching and Breaking of Ultrathin MoS_2_ ,” ACS Nano 5 (2011): 9703–9709, 10.1021/nn203879f. 22087740

[20] M. Li , Q. Zhou , M. Cao , Z. Zhou , and X. Liu , “High‐Temperature Solid Lubrication Applications of Transition Metal Dichalcogenides (TMDCs) MX_2_: A Review,” Nano Materials Science 7 (2025): 409–423, 10.1016/j.nanoms.2024.05.006.

[21] X. Gao , J. Zhang , P. Ju , et al., “Shear‐Induced Interfacial Structural Conversion of Graphene Oxide to Graphene at Macroscale,” Advanced Functional Materials 30 (2020): 2004498, 10.1002/adfm.202004498.

[22] I. Jeon , S. Lee , and S. Yang , “Hyperthermal Erosion of Thermal Protection Nanocomposites Under Atomic Oxygen and N_2_ Bombardment,” International Journal of Mechanical Sciences 240 (2023): 107910, 10.1016/j.ijmecsci.2022.107910.

[23] D. Berman , S. A. Deshmukh , S. K. R. S. Sankaranarayanan , A. Erdemir , and A. V. Sumant , “Macroscale Superlubricity Enabled by Graphene Nanoscroll Formation,” Science 348 (2015): 1118–1122, 10.1126/science.1262024. 25977372

[24] S. Kumari , A. Chouhan , L. Siva Kumar Konathala , et al., “Chemically Functionalized 2D/2D Hexagonal Boron Nitride/Molybdenum Disulfide Heterostructure for Enhancement of Lubrication Properties,” Applied Surface Science 579 (2022): 152157, 10.1016/j.apsusc.2021.152157.

[25] D. Bao , J. Ning , D. Lin , et al., “A Novel High Thermal Conductivity Powder Coating Based on Synergistic Reinforcement of Heat Conduction and Infrared Heat Radiation,” Industrial Chemistry & Materials 4 (2026): 65–77, 10.1039/D5IM00115C.

[26] J. Chen , X. Xu , J. Zhou , and B. Li , “Interfacial Thermal Resistance: Past, Present, and Future,” Reviews of Modern Physics 94 (2022): 025002, 10.1103/RevModPhys.94.025002.

[27] Y. Zhao , C. Luo , J. Zhang , X. Zhang , F. Wang , and C. Bai , “A Strategy of Hexagonal Boron Nitride Endowing Lubricant Oil With Steady Superlubricity,” Applied Surface Science 697 (2025): 163060, 10.1016/j.apsusc.2025.163060.

[28] Y. Zhang , M. Li , Y. Gu , S. Wang , and Z. Zhang , “Preparation of High‐Content Hexagonal Boron Nitride Composite Film and Characterization of Atomic Oxygen Erosion Resistance,” Applied Surface Science 402 (2017): 182–191, 10.1016/j.apsusc.2017.01.071.

[29] Z.‐H. Li , L. Liu , X. You , et al., “Cooperative Enhancement of Mechanical and Tribological Properties Through Tailoring TiN Transition Interface in Boron Nitride Nanosheets Reinforced Copper Composites,” Rare Metals 43 (2024): 5202–5215, 10.1007/s12598-024-02782-x.

[30] H. Ying , Q. Guo , B. Yuan , et al., “High‐Entropy Boride‐Based Composite Ceramics Based on c‐BN Transformation Toughening: Fracture Toughness and High‐Temperature Tribological Properties,” International Journal of Refractory Metals and Hard Materials 136 (2025): 107576, 10.1016/j.ijrmhm.2025.107576.

[31] Y.‐K. Kim , I. J. Lim , H. Lim , et al., “High‐Density Boron Nitride Nanotube Composites via Surfactant‐Stabilized Lyotropic Liquid Crystals for Enhanced Space Radiation Shielding,” Advanced Functional Materials 35 (2025): 10716, 10.1002/adfm.202510716.

[32] S. Chen , H. Fan , Y. Su , et al., “Tough and Damage‐Tolerant Composite Ceramics Enabled by Bioinspired Multiple Architectures,” Materials Today 90 (2025): 169–178, 10.1016/j.mattod.2025.09.002.

[33] Y. Zhao , J. Zhang , F. Ma , et al., “Computational Exploration of Two‐Dimensional Vacancy‐Free Boridene Sheet and Its Derivatives: High Stabilities and the Promise for Hydrogen Evolution Reaction,” Microstructures 4 (2024): 2024032, 10.20517/microstructures.2023.80.

[34] S. Hu , M. Lozada‐Hidalgo , F. C. Wang , et al., “Proton Transport Through One‐Atom‐Thick Crystals,” Nature 516 (2014): 227–230, 10.1038/nature14015. 25470058

[35] P. Z. Sun , Q. Yang , W. J. Kuang , et al., “Limits on Gas Impermeability of Graphene,” Nature 579 (2020): 229–232, 10.1038/s41586-020-2070-x. 32161387

[36] L. H. Li , J. Cervenka , K. Watanabe , T. Taniguchi , and Y. Chen , “Strong Oxidation Resistance of Atomically Thin Boron Nitride Nanosheets,” ACS Nano 8 (2014): 1457–1462, 10.1021/nn500059s. 24400990

[37] J. H. Kim , D. H. Ryu , S. H. Im , J. Jeong , and C. E. Song , “2D Materials and Additives: A Dual Approach to High‐Performance Tin Perovskite Solar Cells,” Microstructures 5 (2025): 2025063, 10.20517/microstructures.2024.192.

[38] Y.‐M. Wu , G.‐Y. Hou , X. Fan , Q.‐Y. Zhu , M.‐J. Cui , and S.‐M. Ren , “Enhanced Atomic Oxygen Erosion Resistance and Wear Resistance of Epoxy Nanocomposites With KH560‐Functionalized h‐BN Nanohybrids,” Rare Metals 44 (2025): 8952–8968, 10.1007/s12598-025-03451-3.

[39] Q. Cai , D. Scullion , W. Gan , et al., “High Thermal Conductivity of High‐Quality Monolayer Boron Nitride and its Thermal Expansion,” Science Advances 5 (2019): aav0129, 10.1126/sciadv.aav0129. PMC6555632 31187056

[40] D. Wu , Z. Zhao , B. Lin , et al., “Probing Structural Superlubricity of Two‐Dimensional Water Transport With Atomic Resolution,” Science 384 (2024): 1254–1259, 10.1126/science.ado1544. 38870285

[41] J. Li , Y. Su , S. Chen , et al., “High‐Entropy Diboride: A Novel High‐Temperature Self‐Lubricating Ceramic With Enhanced Mechanical and Tribological Properties,” Journal of Advanced Ceramics 14 (2025): 9221085, 10.26599/JAC.2025.9221085.

[42] L. Chen , Z. Li , C. Bai , et al., “O‐to‐N Atom Substitution in h‐BN Impedes its Interlayer Slip in Humid Environments,” Small 21 (2025): 09672, 10.1002/smll.202509672. 41201133

[43] K. Zhan , Y. Chen , Z. Xiong , et al., “Low Thermal Contact Resistance Boron Nitride Nanosheets Composites Enabled by Interfacial Arc‐Like Phonon Bridge,” Nature Communications 15 (2024): 2905, 10.1038/s41467-024-47147-1. PMC10994942 38575613

[44] Z. Lin , J. Li , Z. Sun , et al., “Facile Chemical Surface Modification of Boron Nitride Platelets and Improved Thermal and Mechanical Properties of Their Polymer Compounds for 2.5D/3D Packaging Applications,” Composites Science and Technology 256 (2024): 110778, 10.1016/j.compscitech.2024.110778.

[45] A. Zeinedini and M. M. Shokrieh , “Agglomeration Phenomenon in Graphene/Polymer Nanocomposites: Reasons, Roles, and Remedies,” Applied Physics Reviews 11 (2024): 041301, 10.1063/5.0223785.

[46] J. Wang , L. Gong , S. Xi , C. Li , Y. Su , and L. Yang , “Synergistic Effect of Interface and Agglomeration on Young's Modulus of Graphene‐Polymer Nanocomposites,” International Journal of Solids and Structures 292 (2024): 112716, 10.1016/j.ijsolstr.2024.112716.

[47] G. Kresse and J. Furthmüller , “Efficiency of ab‐initio Total Energy Calculations for Metals and Semiconductors Using a Plane‐Wave Basis Set,” Computational Materials Science 6 (1996): 15–50, 10.1016/0927-0256(96)00008-0.

[48] J. P. Perdew , J. A. Chevary , S. H. Vosko , et al., “Atoms, Molecules, Solids, and Surfaces: Applications of the Generalized Gradient Approximation for Exchange and Correlation,” Physical Review B 46 (1992): 6671–6687, 10.1103/PhysRevB.46.6671. 10002368

[49] J. P. Perdew , K. Burke , and M. Ernzerhof , “Generalized Gradient Approximation Made Simple,” Physical Review Letters 77 (1996): 3865–3868, 10.1103/PhysRevLett.77.3865. 10062328

[50] S. Grimme , A. Hansen , J. G. Brandenburg , and C. Bannwarth , “Dispersion‐Corrected Mean‐Field Electronic Structure Methods,” Chemical Reviews 116 (2016): 5105–5154, 10.1021/acs.chemrev.5b00533. 27077966

[51] V. Wang , N. Xu , J.‐C. Liu , G. Tang , and W.‐T. Geng , “VASPKIT: A User‐Friendly Interface Facilitating High‐Throughput Computing and Analysis Using VASP Code,” Computer Physics Communications 267 (2021): 108033, 10.1016/j.cpc.2021.108033.

[52] A. P. Thompson , H. M. Aktulga , R. Berger , et al., “LAMMPS—A Flexible Simulation Tool for Particle‐Based Materials Modeling at the Atomic, Meso, and Continuum Scales,” Computational Physics Communications 271 (2022): 108171.

[53] N. Uene , T. Mabuchi , M. Zaitsu , S. Yasuhara , A. C. T. van Duin , and T. Tokumasu , “Reactive Force Field Molecular Dynamics Studies of the Initial Growth of Boron Nitride Using BCl_3_ and NH_3_ by Atomic Layer Deposition,” The Journal of Physical Chemistry C 128 (2024): 1075–1086, 10.1021/acs.jpcc.3c06704.

[54] Q. Li , J. Wang , B. Zhu , and Y. Sun , “Insight Into the Formation Mechanism of Oxidation Layer on the Amorphous Boron Nanocluster Surface,” Colloids and Surfaces A: Physicochemical and Engineering Aspects 703 (2024): 135374, 10.1016/j.colsurfa.2024.135374.

[55] A. Stukowski , “Visualization and Analysis of Atomistic Simulation Data With OVITO–the Open Visualization Tool,” Modelling and Simulation in Materials Science and Engineering 18 (2010): 015012, 10.1088/0965-0393/18/1/015012.

[56] H. Jiang , J. Li , Y. Xie , et al., “Rapid Exfoliation and Surface Hydroxylation of High‐Quality Boron Nitride Nanosheets Enabling Waterborne Polyurethane With High Thermal Conductivity and Flame Retardancy,” Advanced Composites and Hybrid Materials 7 (2024): 8, 10.1007/s42114-023-00818-x.

[57] S. C. Yoo , J. Kim , W. Lee , J. Y. Hwang , H. J. Ryu , and S. H. Hong , “Enhanced Mechanical Properties of Boron Nitride Nanosheet/Copper Nanocomposites via a Molecular‐Level Mixing Process,” Composites Part B: Engineering 195 (2020): 108088, 10.1016/j.compositesb.2020.108088.

[58] M. G. Rasul , A. Kiziltas , B. Arfaei , et al., “2D Boron Nitride Nanosheets for Polymer Composite Materials,” npj 2D Materials and Applications 5 (2021): 56.

[59] X. Tian , N. Wu , B. Zhang , Y. Wang , Z. Geng , and Y. Li , “Glycine Functionalized Boron Nitride Nanosheets With Improved Dispersibility and Enhanced Interaction With Matrix for Thermal Composites,” Chemical Engineering Journal 408 (2021): 127360, 10.1016/j.cej.2020.127360.

[60] C. Bai , Z. Yang , J. Zhang , B. Zhang , Y. Yu , and J. Zhang , “Friction Behavior and Structural Evolution of Hexagonal Boron Nitride: A Relation to Environmental Molecules Containing −OH Functional Group,” ACS Applied Materials & Interfaces 14 (2022): 19043–19055, 10.1021/acsami.2c02450. 35416641

[61] D. Schmelter and H. Hintze‐Bruening , “Highly Ordered Graphene Oxide and Reduced Graphene Oxide Based Polymer Nanocomposites: Promise and Limits for Dynamic Impacts Demonstrated in Model Organic Coatings,” ACS Applied Materials & Interfaces 8 (2016): 16328–16338, 10.1021/acsami.6b04347. 27254080

[62] V. Ghai , A. A. Mishra , E. Huang , et al., “Halbach Array Induced Magnetic Field Alignment in Boron Nitride Nanocomposites,” Advanced Science 12 (2025): 2408532, 10.1002/advs.202408532. 39716871 PMC11809381

[63] G. Liang , G. Sun , J. Bi , W. Wang , X. Yang , and Y. Li , “Mechanical and Dielectric Properties of Functionalized Boron Nitride Nanosheets/Silicon Nitride Composites,” Ceramics International 47 (2021): 2058–2067, 10.1016/j.ceramint.2020.09.038.

[64] Q. Song , X. Long , B. Wang , et al., “Boron Nitride Nanosheets Composite SiC Fibers With Enhanced Mechanical Properties and High‐Temperature Resistance,” Journal of the American Ceramic Society 108 (2025): 20193, 10.1111/jace.20193.

[65] T. Kusunose , Y. Uno , Y. Tanaka , and T. Sekino , “Isotropic Enhancement of the Thermal Conductivity of Polymer Composites by Dispersion of Equiaxed Polyhedral Boron Nitride Fillers,” Composites Science and Technology 208 (2021): 108770, 10.1016/j.compscitech.2021.108770.

[66] Z. Wang , C. Bian , J.‐C. Zhang , et al., “Plasma‐Modified Boron Nitride Nanosheets for High‐Performance Aramid‐Based Dielectric Films With Enhanced Multifunctionality,” Advanced Science 13 (2026): 16944, 10.1002/advs.202516944. PMC12767117 41133984

[67] L.‐H. Zhao , L. Wang , Y.‐F. Jin , J.‐W. Ren , Z. Wang , and L.‐C. Jia , “Simultaneously Improved Thermal Conductivity and Mechanical Properties of Boron Nitride Nanosheets/Aramid Nanofiber Films by Constructing Multilayer Gradient Structure,” Composites Part B: Engineering 229 (2022): 109454, 10.1016/j.compositesb.2021.109454.

[68] A. Muthurasu , R. Balaji , T. H. Ko , et al., “Atomically Dispersed Iron on Functionalized Boron Nitride Nanosheets for Efficient Oxygen Reduction in Proton Exchange Membrane Fuel Cells,” ACS Catalysis 15 (2025): 18987–18994, 10.1021/acscatal.5c06792.

[69] Y. Fouchal , R. Ramirez , M. Beloreshka , and E. A. Plis , “Comparative Evaluation of Spacecraft Materials Properties Under Simulated and True Space Environments,” The Journal of the Astronautical Sciences 71 (2024): 53, 10.1007/s40295-024-00476-1.

[70] L. Xing , Q. Li , G. Zhang , et al., “Self‐Healable Polymer Nanocomposites Capable of Simultaneously Recovering Multiple Functionalities,” Advanced Functional Materials 26 (2016): 3524–3531, 10.1002/adfm.201505305.

[71] D. Yang , L. Zhang , T. Yu , et al., “Hierarchical MoS2‐Oleogel in Porous Polyimides: A Self‐Adaptive Confined Lubrication Strategy for Ultralow Friction and Wear,” Advanced Functional Materials 35 (2025): 202517029.

