# Supplementary material for: Bridged h‐BN Nanosheets Coatings: Simultaneous Shielding Atomic‐Oxygen Irradiation and Achieving Superior Friction Performance
Source: Adv Sci (Weinh). 2026 Jun 12:e76093. Online ahead of print. doi: 10.1002/advs.76093 (PMC13337121; doi:10.1002/advs.76093)
Supplement: Supplementary file 1 — Supporting File: advs76093‐sup‐0001‐SuppMat.docx. [file ADVS-9999-e76093-s001.docx]

**Supplementary Information**

**Bridged h-BN Nanosheets Coatings: Simultaneous Shielding Atomic-Oxygen Irradiation and Achieving Superior Friction Performance**

Zhuoyi Li ^1, 2^, Changning Bai ^1,*^, Xingkai Zhang ^2^, Yusheng Liang ^3^, Wei Wu ^1^,

Dingrui Zhou ^2^, Tao Du ^4,*^, Chunjin Wang ^1,*^

**^1^** State Key Laboratory of Ultra-precision Machining Technology, Department of Industrial and Systems Engineering, The Hong Kong Polytechnic University, Hong Kong 999077, China.

**^2^** State Key Laboratory of Solid Lubrication, Lanzhou Institute of Chemical Physics, Chinese Academy of Sciences, Lanzhou 730000, China.

**^3^** College of Civil Engineering and Mechanics, Lanzhou University, Lanzhou 730000, China.

**^4^** Key Lab of Smart Prevention and Mitigation of Civil Engineering Disasters of the Ministry of Industry and Information Technology, Harbin Institute of Technology, Harbin, 150090, China.

Corresponding authors:

Changning Bai, E-mail: [changning.bai@polyu.edu.hk](mailto:changning.bai@polyu.edu.hk)

Tao Du, E-mail: dutao@hit.edu.cn

Chunjin Wang, E-mail: [chunjin.wang@polyu.edu.hk](mailto:chunjin.wang@polyu.edu.hk)

**Table S1**

Table S1 Formulation details of Films A – C

| Component | Film A | Film B | Film C |
| --- | --- | --- | --- |
| PVA | 10 wt% | 10 wt% | 10 wt% |
| AAm | 5 wt% | 5 wt% | 5 wt% |
| MBAA  APS  Glutaraldehyde  BNNS@AAm | 0.5 wt%  0.1 wt%  0.5 wt%  0 wt% | 0.5 wt%  0.1 wt%  0.5 wt%  0.093 wt% | 0.5 wt%  0.1 wt%  0.5 wt%  0.186 wt% |

**Figure S1**


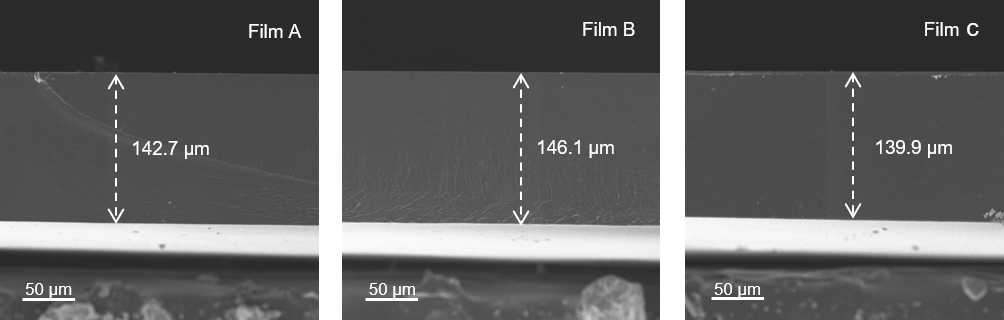


**Figure S1.** Cross-sectional SEM images of Film A, Film B, and Film C.

**Figure S2**


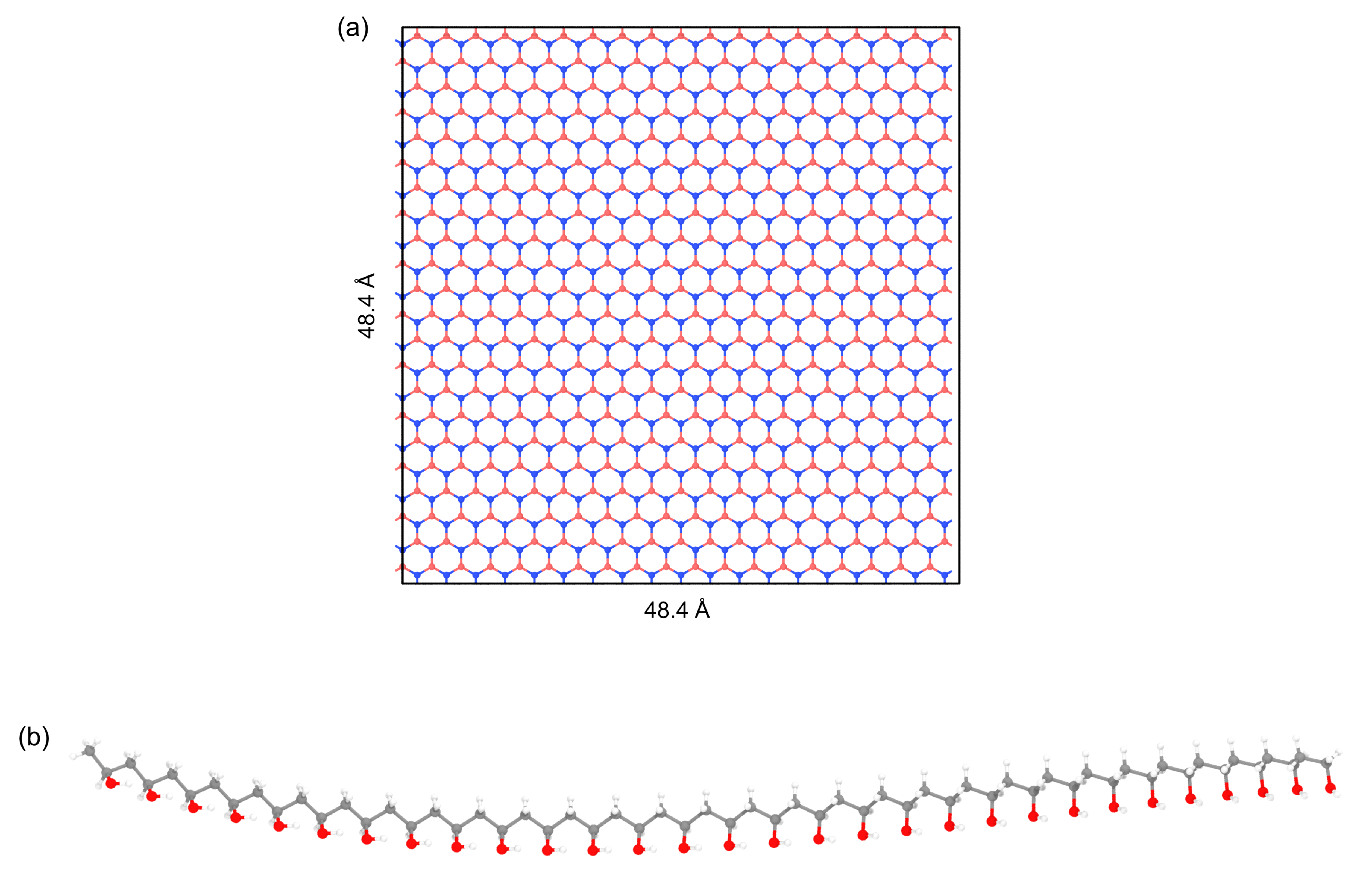
**Figure S2.** Schematics of the basic units of (a)h-BN and (b) PVA.

**Figure S3**

**
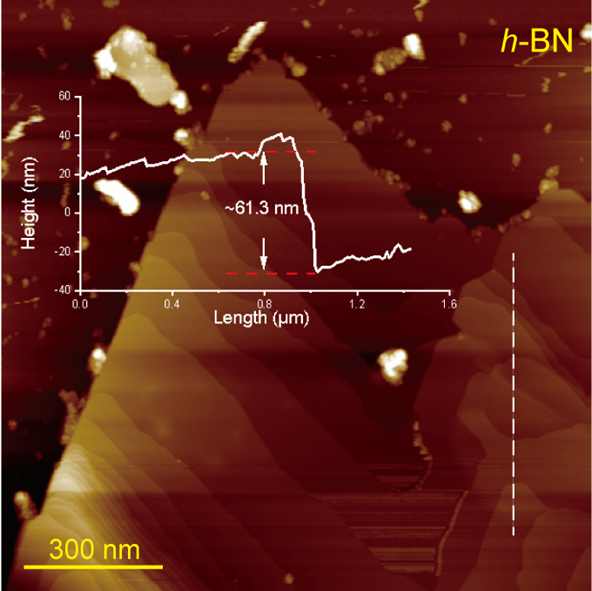
**

**Figure S3.** AFM image of h-BN.

**Figure S4**


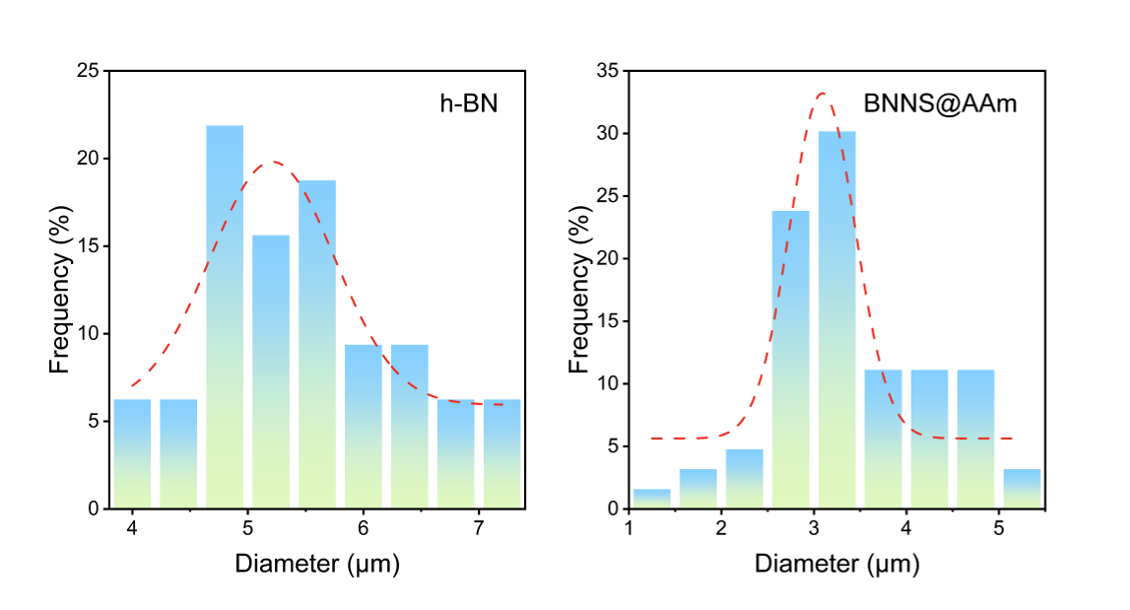


**Figure S4.** AFM-based statistical distributions of the lateral diameter of raw h-BN and exfoliated BNNS@AAm. The average lateral diameters were about 5.2 μm for h-BN and 3.1 μm for BNNS@AAm, respectively.

**Figure S5**

**
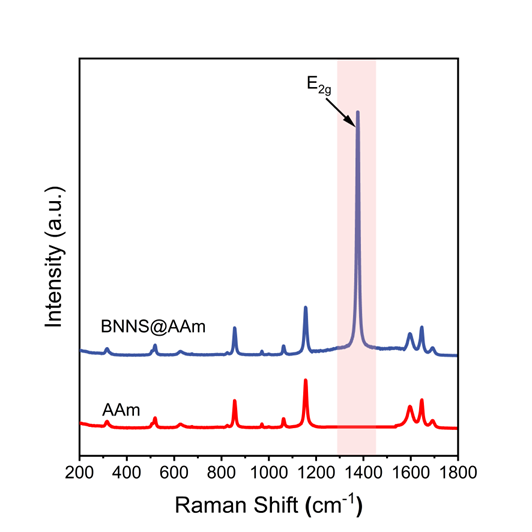
**

**Figure S5.** Raman spectra of the as-prepared BNNS@AAm and AAm.

**Figure S6**

**
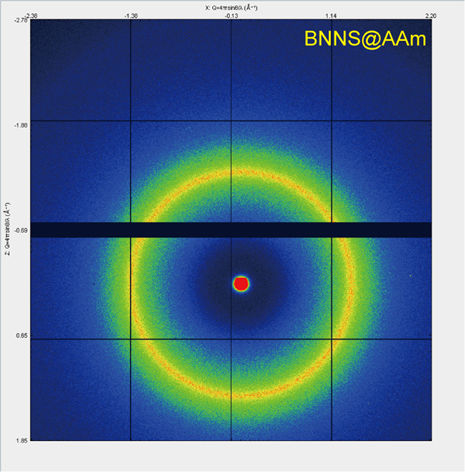
**

**Figure S6.** SAXS diffraction pattern of the as-prepared BNNS@AAm used for film fabrication.

**Figure S7**

**
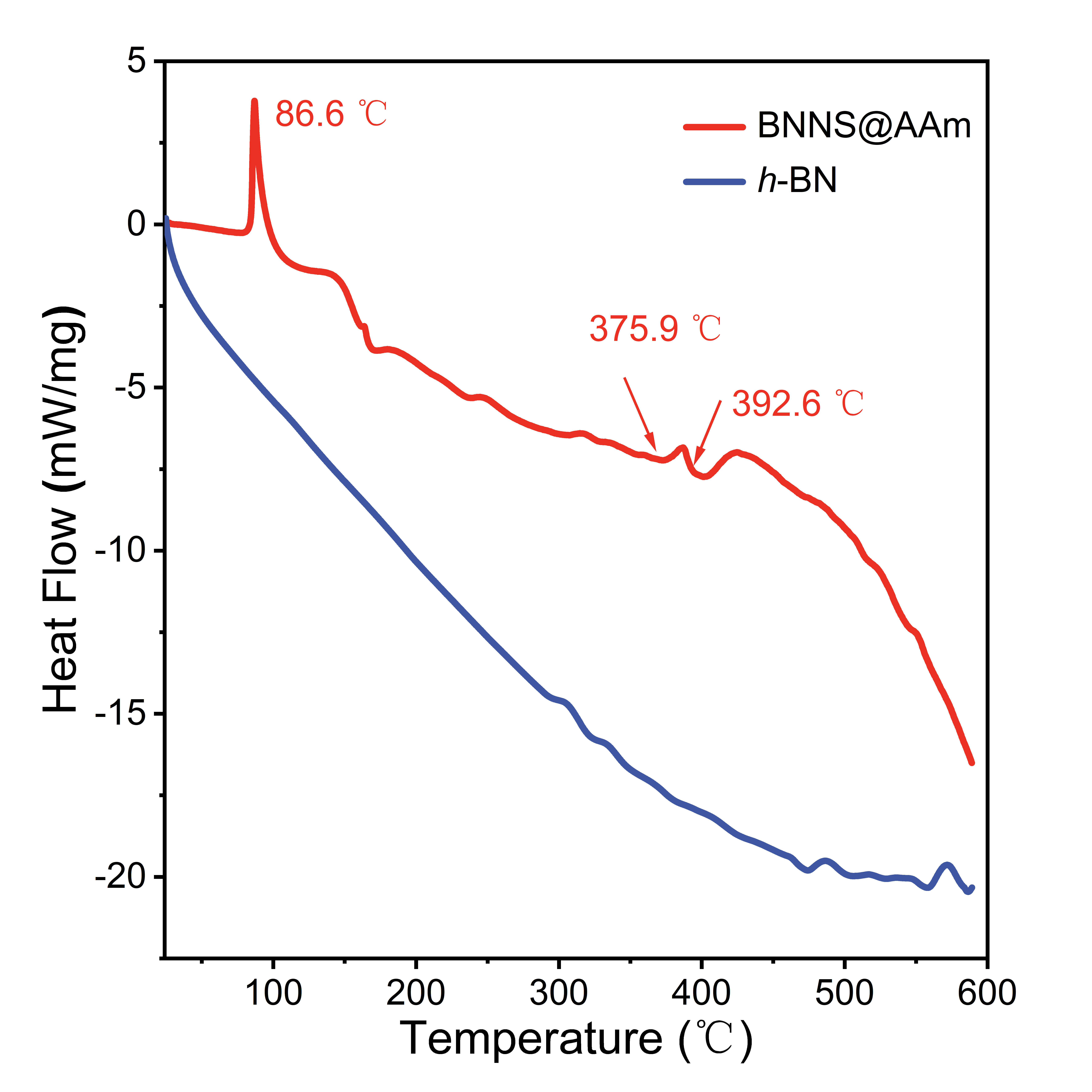
**

**Figure S7.** DSC curve of the as-prepared BNNS@AAm.

**Figure S8**


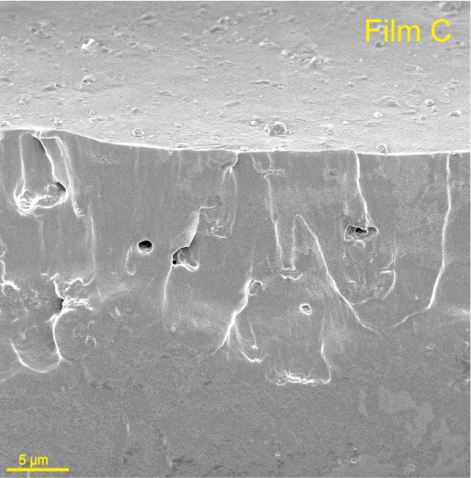

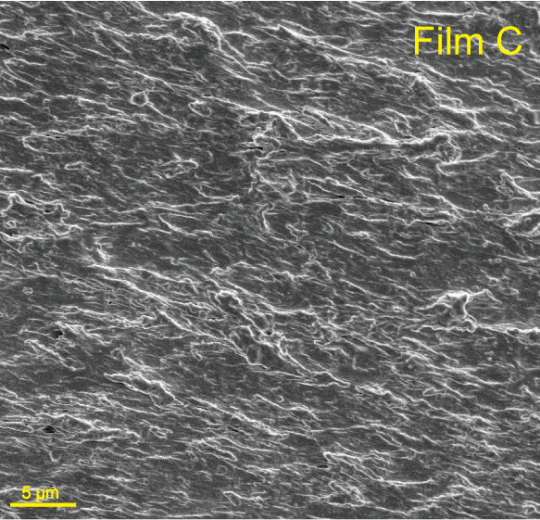


**Figure S8.** SEM image of the cross-section and the surface of Film C.

**Figure S9**


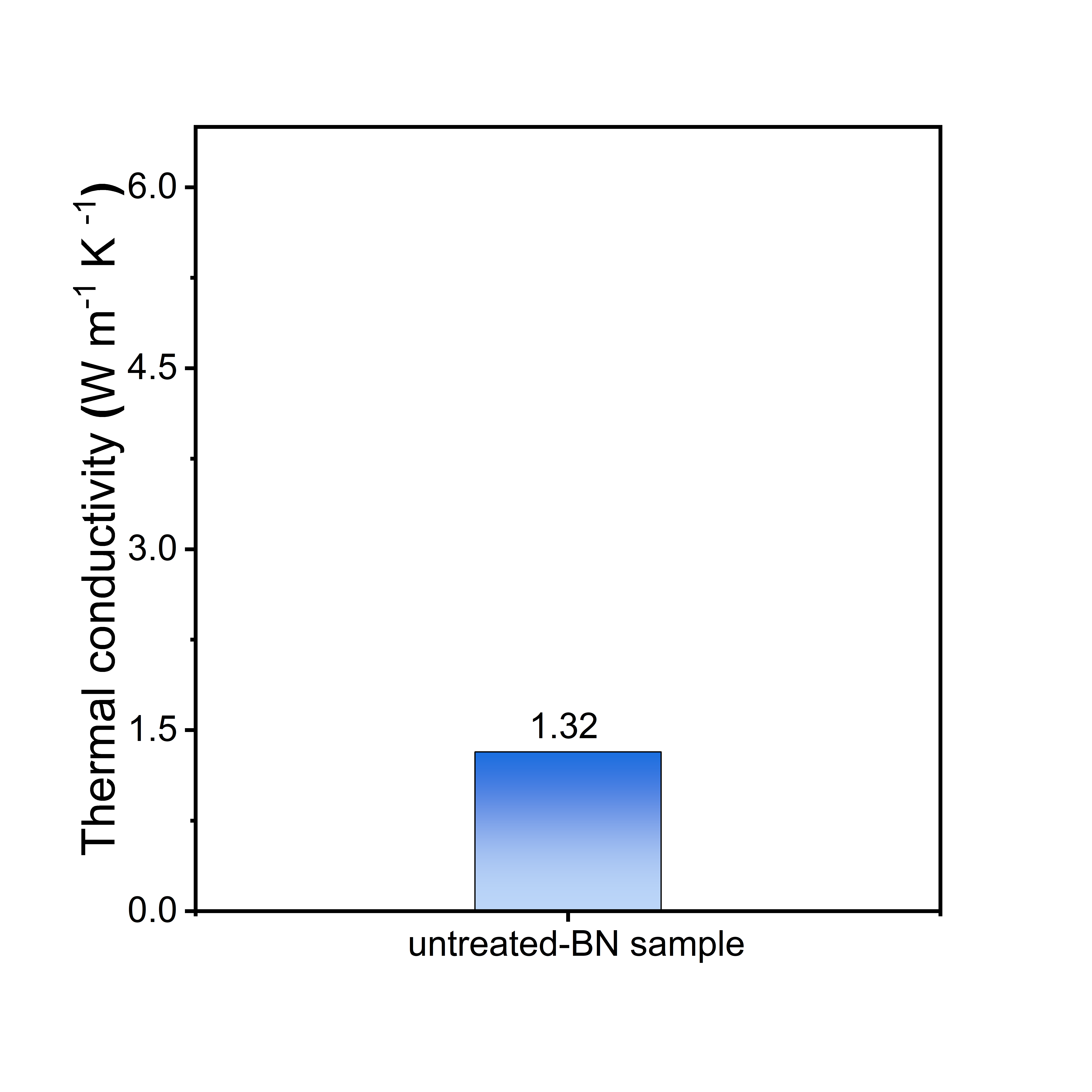


**Figure S9.** Thermal conductivity of the untreated-BN sample measured under otherwise identical conditions.

**Figure S10**


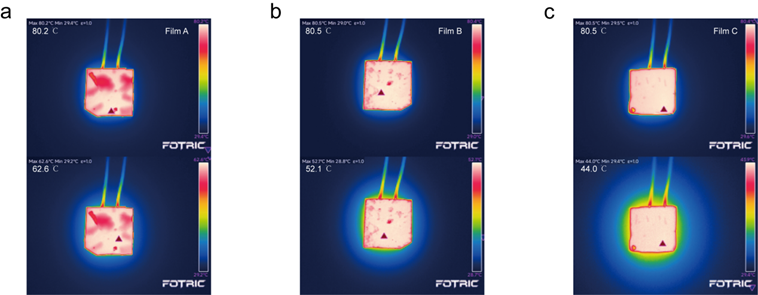


**Figure S10.** The infrared thermal imaging images of Film A, Film B and Film C.

**Figure S11**


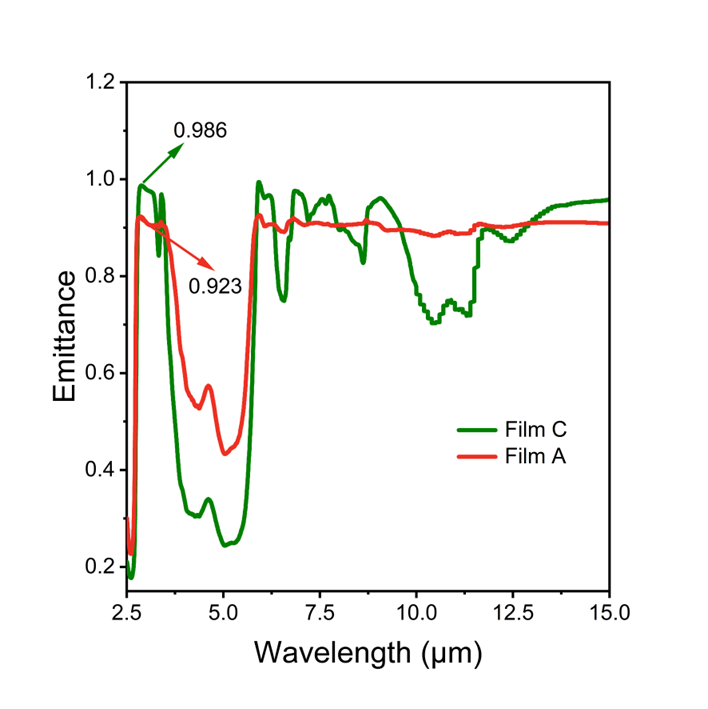


**Figure S11.** The infrared emissivity curves of film A and film C.

**Figure S12**


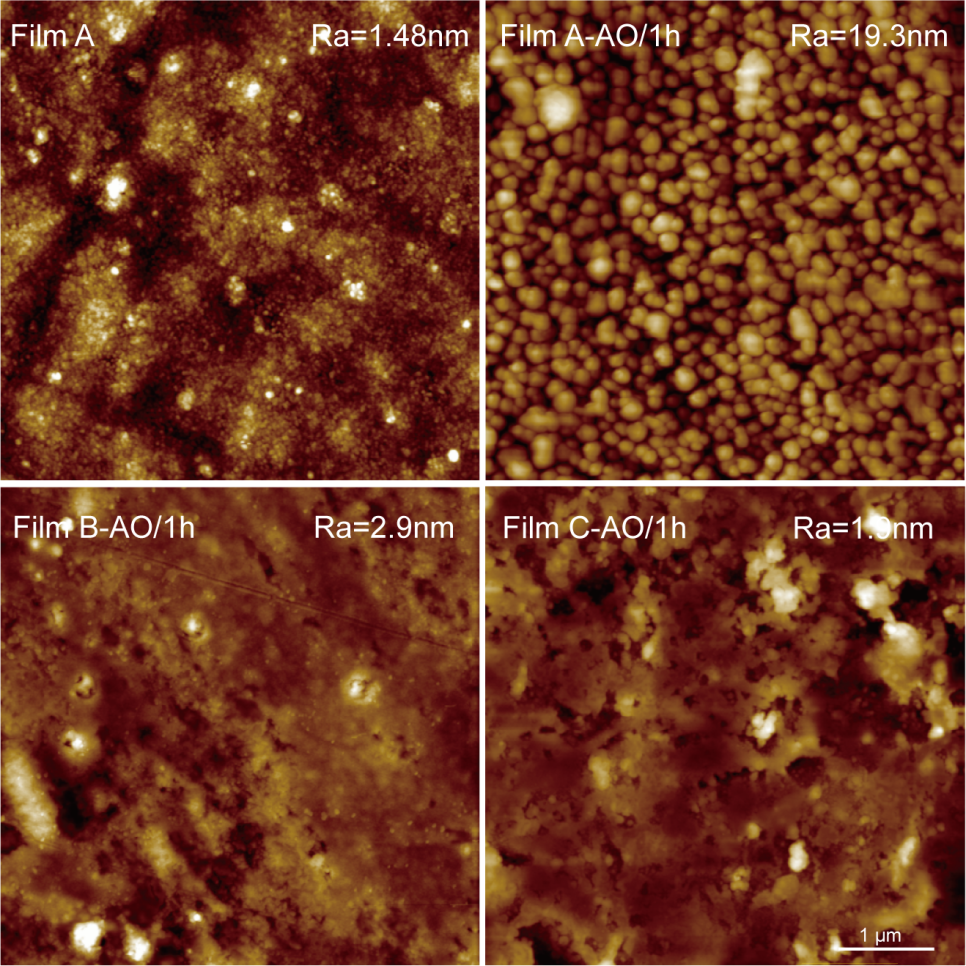


**Figure S12.** Large-scale **AFM images of Film A and Films A, B, and C after 1 h of AO irradiation.**

**Figure S13**


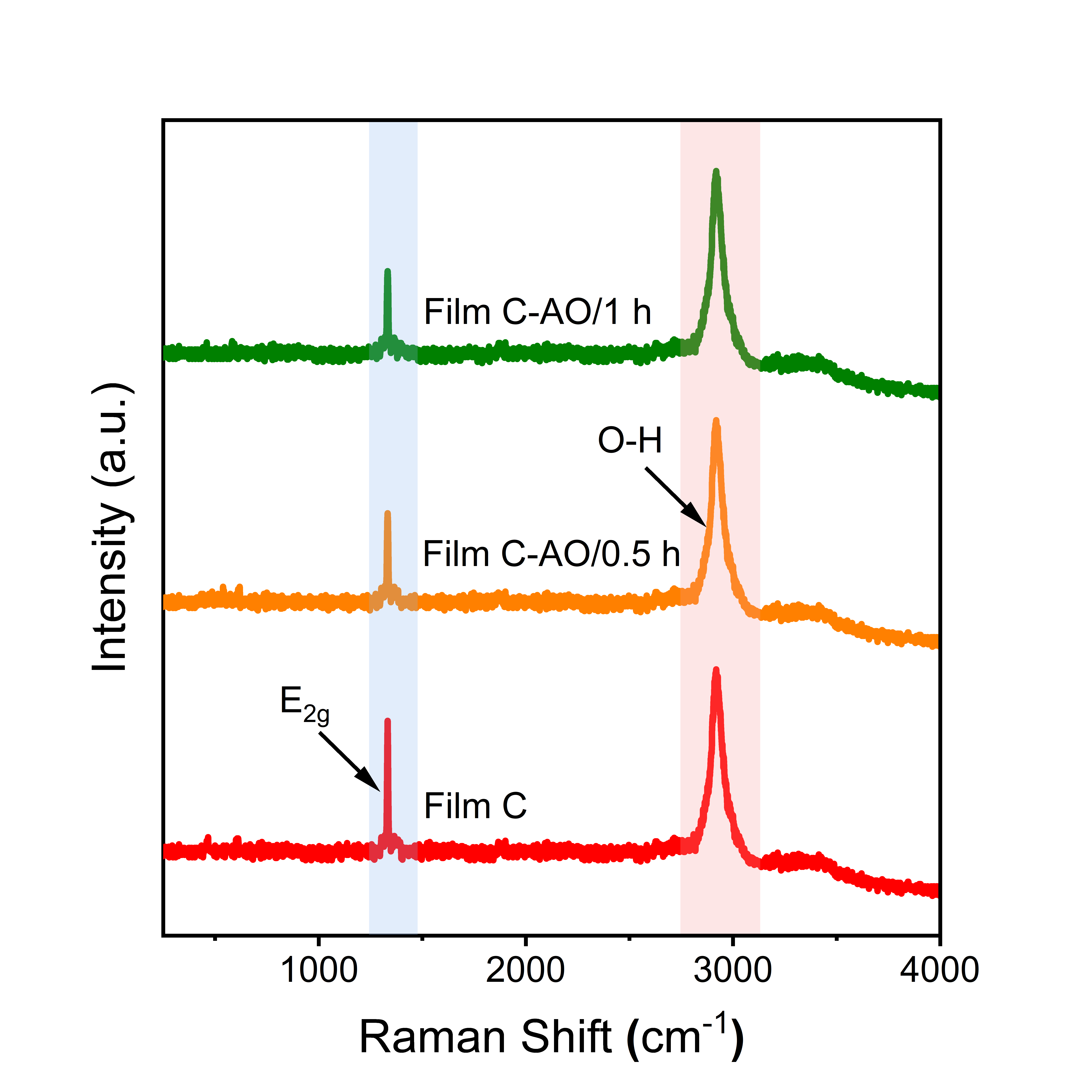


**Figure S13.** Raman spectr**a of Film C after no irradiation, 0.5 h AO irradiation, and 1 h AO irradiation.**

**Figure S14**


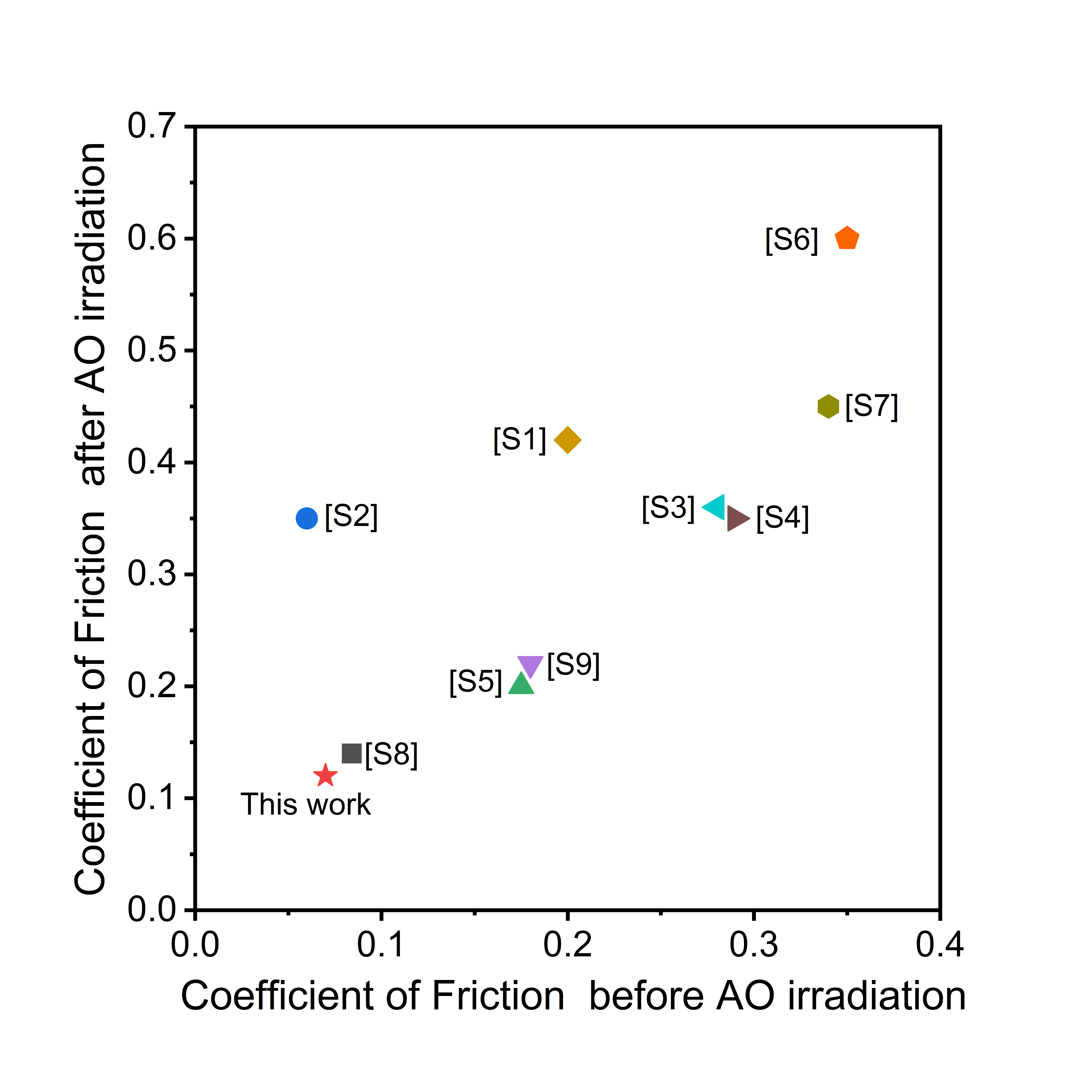


**Figure S14.** Comparison of the coefficients of friction of representative systems before and after AO irradiation. The present work is located in the lower-left region, indicating relatively low friction under both conditions.

References:

[S1] H. Qi, Y. Lei, X. Lei, et al., “High Wear Resistance of POSS Grafted-Polyimide/Silica Composites under Atomic Oxygen Conditions,” Polymers 15 (2023): 2385.

[S2] C. Yu, P. Ju, H. Wan, et al., “Enhanced atomic oxygen resistance and tribological properties of PAI/PTFE composites reinforced by POSS,” Progress in Organic Coatings 139 (2020): 105427.

[S3] M. Lv, Y. Wang, Q. Wang, et al., “Effects of individual and sequential irradiation with atomic oxygen and protons on the surface structure and tribological performance of polyetheretherketone in a simulated space environment,” RSC Advances 5 (2015): 83065-83073.

[S4] G. Zhao, Q. Ding, Q. Wang, et al., “Ultraviolet or Atomic Oxygen Effects on Tribological Properties of the Carbon Fibers/Polyimide Composites,” Journal of Macromolecular Science, Part B: Physics 54 (2015): 593-604.

[S5] C. Han, G. Li, G. Ma, et al., “Research on Atomic Oxygen Erosion Influence of Structural Damage and Tribological Properties of Mo/MoS_2_-Pb-PbS Thin Film,” Materials 15 (2022): 1851.

[S6] Y. Liu, J. Yang, Z. Ye, et al., “Influence of Atomic Oxygen Exposure on Friction Behavior of 321 Stainless Steel,” Protection of Materials and Structures From the Space Environment, Astrophysics and Space Science Proceedings 32 (2013): 371-379.

[S7] Y. Liu, Z. Ye, J. Yang, et al., “Effects of Atomic Oxygen Exposure on Tribological Property of Zirconium Alloy,” Protection of Materials and Structures from the Space Environment, Astrophysics and Space Science Proceedings 47 (2017): 293-302.

[S8] X. Liu, L. Wang, Q. Xue, “High vacuum tribological performance of DLC-based solid-liquid lubricating coatings: Influence of atomic oxygen and ultraviolet irradiation,” Tribology International 60 (2013): 36-44.

[S9] Y. Si, J. Guo, J. Liu, et al., “Effects of simulated space atomic oxygen irradiation on the lubrication properties and mechanisms of gallium-based liquid metal,” Wear 576-577 (2025): 206132.

**Figure S15**


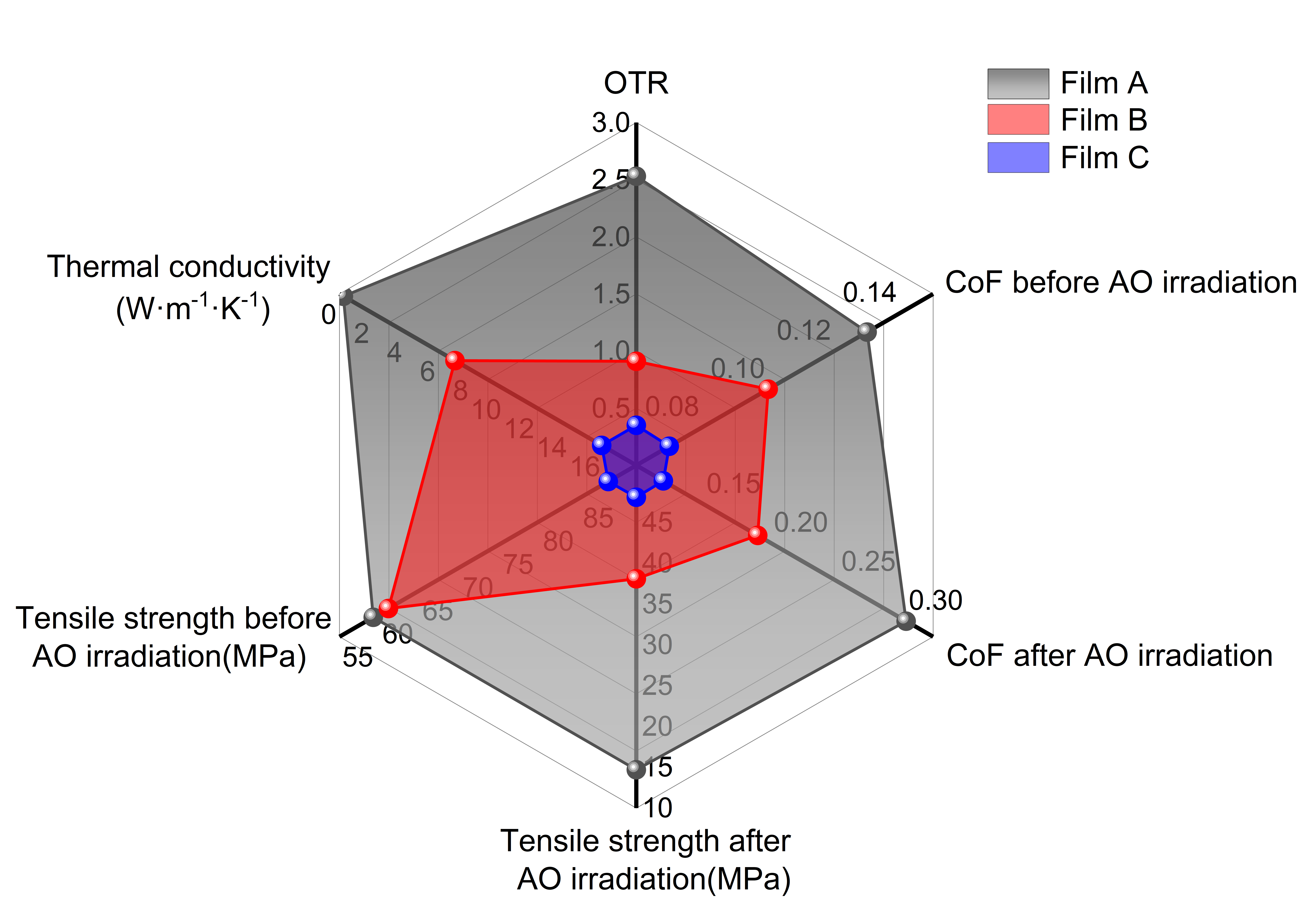


**Figure S15.** Radar-chart comparison of six representative performance parameters of Films A, B, and C, including OTR, friction coefficient before AO irradiation, friction coefficient after AO irradiation, tensile strength before AO irradiation, tensile strength after AO irradiation, and thermal conductivity, highlighting the overall multifunctional performance differences among the three films.

**Figure S16**


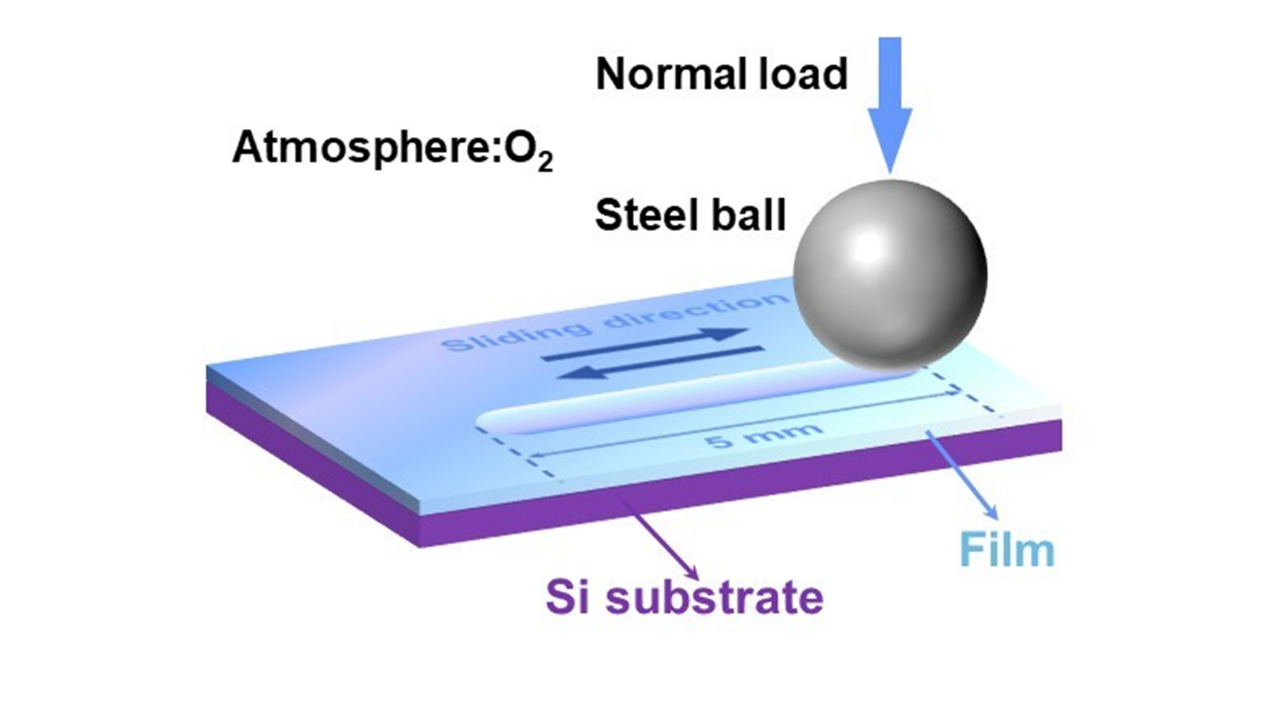


**Figure S16.** The schematic of the tribological experiment.

**Figure S17**


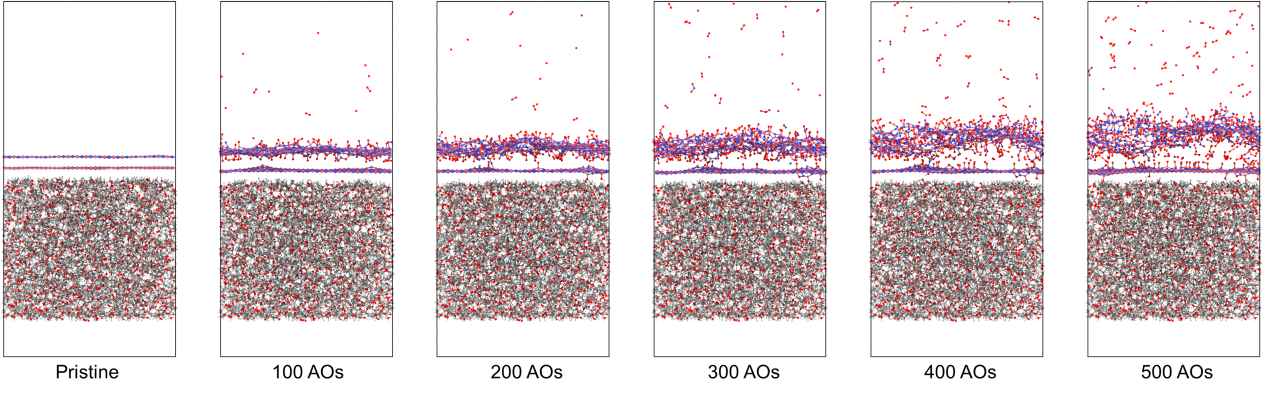


**Figure S17.** Atomic snapshots of PVA with bilayer h-BN under sustained AO exposure.

**Figure S18**


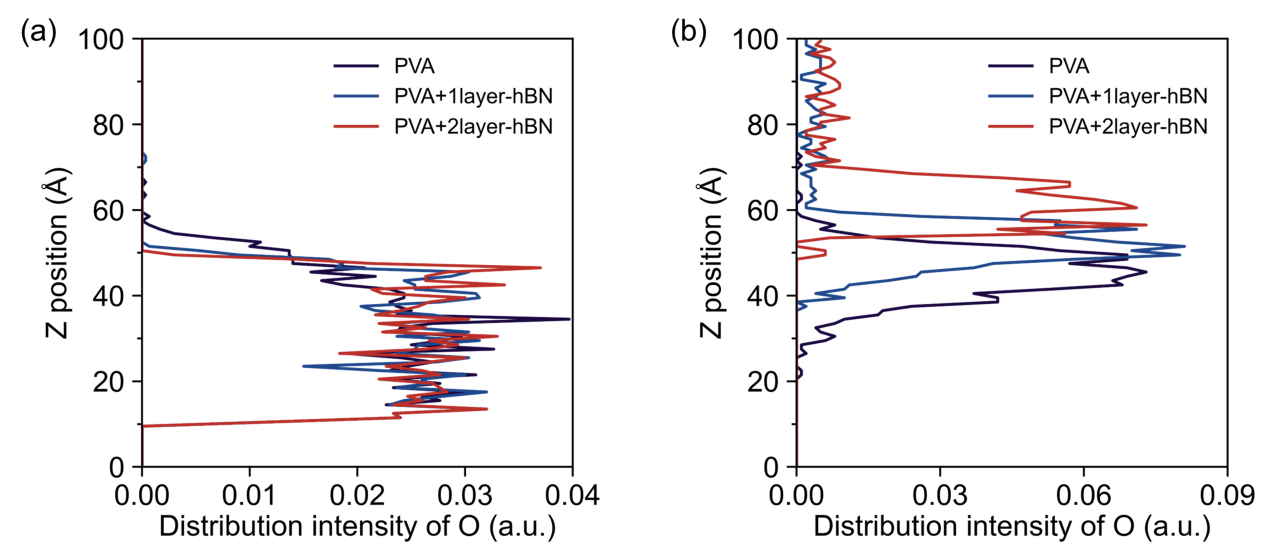


**Figure S18.** The distribution of O from (a) PVA and (b) AOs for different systems after 500 AOs explosure.

**Figure S19**


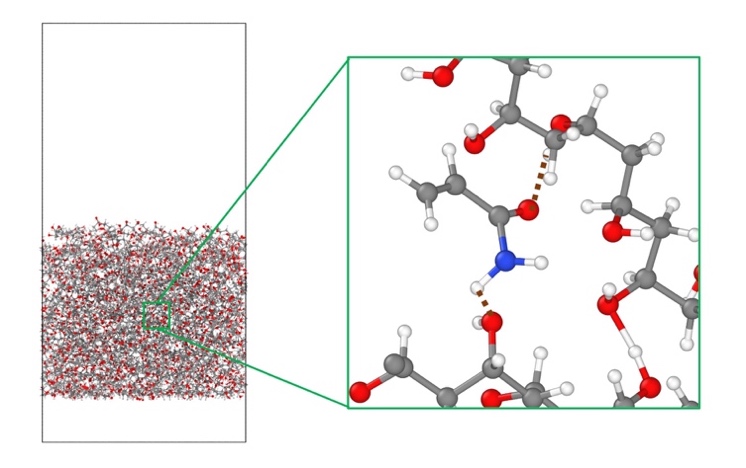


**Figure S19.** Atomic snapshots of the PVA-AAm mixed structure, with a partially magnified view illustrating the hydrogen-bonding interactions between PVA and AAm.

**Figure S20**


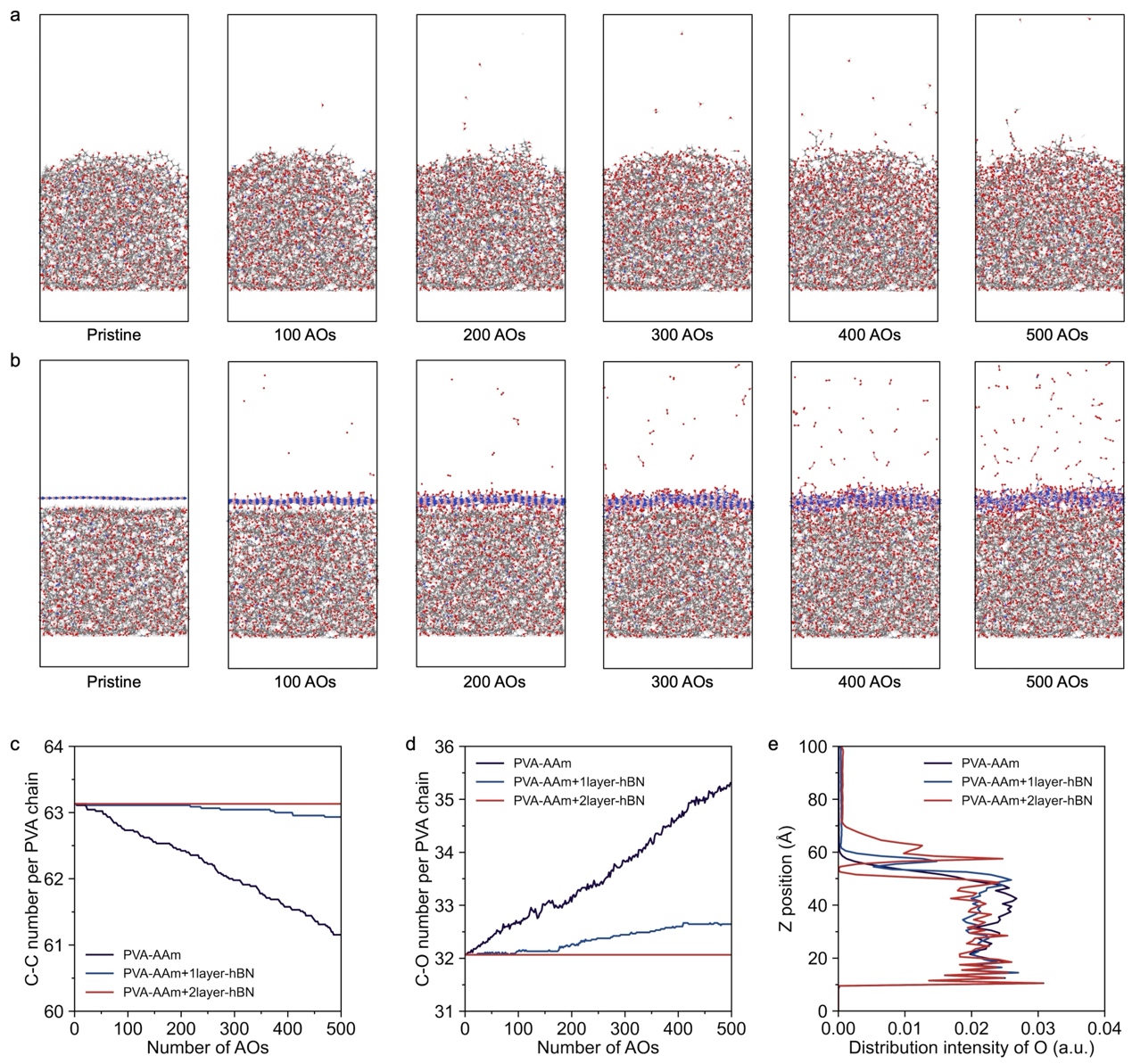


**Figure S20.** Atomic snapshots of (a) PVA-AAm and (b) PVA-AAm with a monolayer h-BN under sustained AO exposure. (c) Number of C–C bonds and (d) number of C–O bonds as a function of AO exposure. (e) Spatial distribution of O atoms in different systems after exposure to 500 AO species.

**Figure S21**


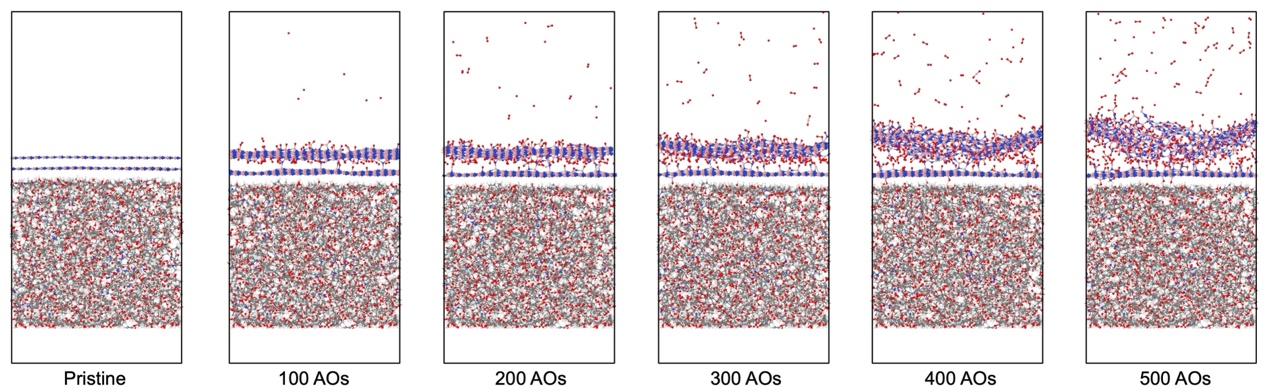


**Figure S21.** Atomic snapshots of PVA-AAm with bilayer h-BN under sustained AO exposure.
